# High resolution global spatiotemporal assessment of rooftop solar photovoltaics potential for renewable electricity generation

**DOI:** 10.1038/s41467-021-25720-2

**Published:** 2021-10-05

**Authors:** Siddharth Joshi, Shivika Mittal, Paul Holloway, Priyadarshi Ramprasad Shukla, Brian Ó Gallachóir, James Glynn

**Affiliations:** 1 SFI MaREI Centre for Energy Climate and Marine, Cork, Ireland; 2 Environmental Research Institute, University College Cork, Cork, Ireland; 3 School of Engineering, University College Cork, Cork, Ireland; 4 Grantham Institute–Climate Change and the Environment, Imperial College London, London, United Kingdom; 5 Department of Geography, University College Cork, Cork, Ireland; 6 Global Centre for Environment and Energy, Ahmedabad University, Ahmedabad, India; 7 Center on Global Energy Policy, Columbia University, New York, USA

**Keywords:** Energy and society, Energy modelling, Energy access, Energy policy, Energy supply and demand

## Abstract

Rooftop solar photovoltaics currently account for 40% of the global solar photovoltaics installed capacity and one-fourth of the total renewable capacity additions in 2018. Yet, only limited information is available on its global potential and associated costs at a high spatiotemporal resolution. Here, we present a high-resolution global assessment of rooftop solar photovoltaics potential using big data, machine learning and geospatial analysis. We analyse 130 million km^2^ of global land surface area to demarcate 0.2 million km^2^ of rooftop area, which together represent 27 PWh yr^−1^ of electricity generation potential for costs between 40–280 $ MWh^−1^. Out of this, 10 PWh yr^−1^ can be realised below 100 $ MWh^−1^. The global potential is predominantly spread between Asia (47%), North America (20%) and Europe (13%). The cost of attaining the potential is lowest in India (66 $ MWh^−1^) and China (68 $ MWh^−1^), with USA (238 $ MWh^−1^) and UK (251 $ MWh^−1^) representing some of the costliest countries.

## Abstract (web summary)

Though a global assessment of rooftop solar photovoltaic (RTSPV) technology’s potential and the cost is needed to estimate its impact, existing methods demand extensive data processing. Here, the authors report a machine learning method to realize a high-resolution global assessment of RTSPV potential.

## Introduction

From powering the National Aeronautics and Space Administration (NASA’s) Vanguard satellites in 1958 to lighting homes in sub-Saharan Africa, solar photovoltaics (PV) technology has come a long way. Rooftop Solar photovoltaics (RTSPV) technology as a subset of the solar photovoltaic electricity generation portfolio can be deployed as a decentralized system either by individual homeowners or by large industrial and commercial complexes. Over the past decade, reduction in the deployment cost coupled with policy-driven initiatives has led to a rapid uptake of RTSPV globally. Between 2006 and 2018, the installed capacity of the RTSPV has grown from 2.5 GW to 213 GW- an 85-fold increase globally^1^. With an additional capacity installation of 41 GW, RTSPV currently accounts for 40% of the global cumulative installed capacity of the solar PV and nearly one-fourth of the total renewable capacity additions in 2018—surpassing the combined new installed capacities of both coal and nuclear. At the same time, RTSPV technology has demonstrated a steep decline in its deployment costs which ranged between 63 and 265 $ MWh^−1^ in the year 2019—a reduction of between 42 and 79% over 2010 values^2^.

Globally, nearly 800 million people were without electricity in 2018, the majority of who are living in rural areas^3^. Here, the role of decentralized rooftop PV in advancing the ethos of the Sustainable Development Goal (SDG) 7 becomes very important. The fast installation time and low levelised cost of RTSPV can aid in mitigating the problem of energy access by making citizens or communities a prosumer. The prosumer can generate and consume electricity as per their requirements without depending exclusively on a centralized grid infrastructure. As the fastest deployable energy generation technology with the highest year-on-year growth rate^4^, solar PV technology is projected to supply 25–49% of the global electricity needs by 2050 while providing employment for up to 15 million people between 2018 and 2050^5^. Out of this, RTSPV deployment will contribute up to 40% of the total solar PV-derived electricity generation by 2050.

Increased deployment of RTSPV can support displacing fossil fuels out of the current energy generation mix as can be observed in the successful implementation of rooftop photovoltaics in Germany. As the demand for electricity as an energy source increases in the future, RSTPV based generation sources will form a large part of the future renewable-based generation portfolio. This shift in the current generation mix coupled with the future low carbon generation capacity expansion can aid in reducing the energy-derived greenhouse gas emissions and also aid in advancing the SDG13 goal of combating climate change with co-benefits for the SDG3. RTSPV technology can thus lead to consumer-driven breakthroughs in tackling climate change, reducing local air pollution, accelerating development, and providing affordable energy access to areas lacking electrification.

To better understand the role that an RTSPV system can fulfill in the future, a global harmonized geo-mapped assessment of its technical potential and the costs associated with attaining the technical potential is pertinent especially when such assessments at a global level are lacking. RTSPV systems are deployed as a decentralized system contrary to the utility-scale solar PV systems, which increases the complexity of its assessment as the smallest unit of deployment becomes a rooftop as opposed to a large plot of a green or brownfield site. Along with the complexities associated with accurately determining the rooftop area, assessment of seasonal variations of its potential is also important to understand the supply dynamics of variable renewable energy (VRE) technologies like RTSPV. This highlights the need for a high-resolution spatiotemporal assessment that accurately represents the geographical variability of the built environment along with impacts of seasonal changes in solar insolation.

Current research primarily focuses on utility-scale solar PV resource assessment at a global scale. A similar assessment has not been done for decentralized RTSPV at a scale greater than regional/national levels^6–9^. As a result, energy system models and research informing climate change policy have not fully considered the role of solar PV in meeting the climate change mitigation goals^10^. Assessment of RTSPV potential requires an underlying dataset of building footprints, solar insolation mapping, and technology-specific information like panel size, conversion efficiency, and system losses. The current literature is adequate in providing global information on the latter two categories, with the largest inaccuracies^11^ attributed to the demarcation and calculation of building footprints which require large data and costly information processing hardware to extract buildings from satellite imagery^12^.

Two major approaches are currently used to determine built-up area, or more specifically the extent and area occupied by building rooftops, Supplementary Table 7. The first approach addresses the problem from a “Bottom–up”^13–23^ perspective and is the most common approach currently implemented to calculate rooftop area at scale. Such approaches establish the relationship between building footprint data (cadastral, crowd-sourced, satellite-derived) and socio-economic metrics (Gross Domestic Product (GDP), Population) for a small sample set and then estimate the extent of building footprints across a wider scale. In the study by Jacobson et al.^24^, the authors have used the bottom-up methodology to calculate available rooftop areas for 179 countries by establishing relationships between population, GDP, and floorspace area per capita based on sample data from the USA and several European countries. For a full set of global countries, Gernaat et al.^25^ have used the relationship between population density, household expenditure, and rooftop area to calculate available rooftop area per country. They have calibrated their relationship equations on World Bank’s data and realized an *R*^2 ^= 0.66. These methods are quick to implement and are relatively accurate when predicting building footprint areas in the neighborhood regions, but inaccuracies arise when the analysis is upscaled towards country/regional levels. This reduction in accuracy^26^ can be attributed to the inaccuracies in the coarse geospatial mapping of socioeconomic data^27^ and the heterogeneity of built-up landscapes.

The second approach addresses the problem from a “Top–Down”^28–39^ perspective, utilizing aerial imagery to determine built-up area along with building footprints contained in it. “Top–Down” methods include earth observation, drone-mounted Light Detection and Ranging (LiDAR), and machine learning (ML) classification algorithms to detect buildings. On a country-wide scale, Gagnon et al.^7^ have used LiDAR datasets for 128 US sample cities to calculate available rooftop areas for the continental USA using statistical inferencing to extrapolate beyond their sample site. They have generated statistical measures to generalize rooftop orientation, slope, and availability based on high-resolution LiDAR imagery. Collecting, processing, and analyzing the aerial imagery is a costly and computationally intensive task requiring datacentre scale infrastructure. These shortcomings have led to limitations in the scale of studies using top-down methods, where only a few commercial^40–42^ ventures have been able to provide country-wide building footprints, and to date, a global-scale analysis has yet to be implemented. One solution to mitigate the processing and data bottleneck is to use predefined land cover classification^43–49^ to demarcate global land areas which are artificially built up and then downscale the built-up area to actual building footprint^49^ using simplifying assumptions. This hybrid approach was used in a study by Bodis et al.^6^, where they have established a relationship between cadastral data of Netherlands, CORINE landcover, and European Settlement Map to infer the rooftop area in the other 27 EU countries.

The current state of art^50–55^ methods utilize ML-based object detection algorithms to map individual buildings for Region of Interest (ROI) at the city/country level. However, none of the state of art methods have been applied to a global ROI assessment partly due to the amount of data processing required in ML-based methods and partly due to the limitations of the ML algorithm in detecting similar objects that deviate from training sample sets.

**Fig. 1:**
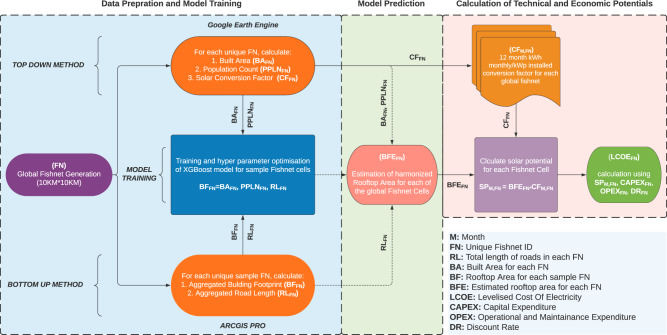
Framework for assessment of RTSPV’s global technical potential and costs. The framework developed in this study starts with data preparation and mapping of various geospatial metrics to both Top-down and Bottom-up pathways. Further, the machine learning model is trained and used to estimate BFE_FN_ values from BA_FN,_ PPLN_FN_, and RL_FN_ values. Next, the BFE_FN_ values are used to calculate the technical potential (SP) with the aid of the conversion factors (CF). Finally, the calculated potential dataset is used to map the Levelised Cost of Electricity (LCOE) values using IRENA’s renewable power generation cost data. The detailed framework is described in the “Results” and “Methods” sections. Model parameters and regional mappings are provided in Supplementary Tables 6, 8–10 and Supplementary Fig. 3a–d.

Here, we directly address the gap in the current literature by developing a hybrid framework that integrates “Top-down” and “Bottom-up” methods using a ML model to provide a high-resolution global assessment of rooftop solar PV technical potential at a monthly temporal resolution (Fig. 1). We divide the entire global landmass into multiple 10 km^2^ assessment units. In each assessment unit, we utilize a high-resolution population dataset (at 100 m spatial resolution), road length, and built-up area boundaries to estimate the rooftop area. The estimation algorithm built upon the ML model is designed to learn the relationship between population, road length and built-up area boundaries, and actual rooftop area from a large global sample set covering countries in various stages of socio-economic development and built-up topography. This way we utilize a comprehensive set of disparate built environment archetypes involving variable population densities in different landscape configurations to overcome the limitations imposed by the “Top-down” and “Bottom-up” methods used in previous studies. Along with the geo-mapping of the technical potential, we also map the associated Levelised Cost of Energy (LCOE) and regional supply cost curves for a global ROI covering >195 countries, spanning 130 million km^2^ land area, containing buildings ranging from detached rural nucleated settlements to global conurbation dotted by multi-storied skyscrapers across varied geographies.

In our study, we define the “technical potential” of RTSPV as the maximum electricity generation that can be derived from a given rooftop area, where the rooftop area is kept consistent with the 2015 year’s built-up extent and does not include additional building stock created after that. The results presented in this study have considered a 100% rooftop availability at a 10% panel efficiency. To account for the change in the potential due to different panel efficiencies and rooftop availability, we have documented global and regional potentials for a set of rooftop scaling factors and panel efficiencies (Supplementary Tables 4 and5). Additional boundary conditions of our study and the limitations in the interpretation of the main results are documented in the “Methods” section.

## Results

### Model design and validation

We started by dividing the global landmass into a Fishnet Grid (FN) containing a total of 3,521,120 unique squares of 10 km^2^ size, where each FN has a unique id and is attributed to a unique country. Aggregation of the built area (BA_FN_) within each FN was undertaken using built area sub-classifications (100 m resolution) provided by the global landcover layer^46^ (LC) of the Copernicus land monitoring program. Being derived from a native 10 m resolution satellite imagery, the LC layer provided us with a significantly improved representation of built-up area over the current state of art methods that utilize coarser-resolution landcover classification. The use of pre-built landcover classifications also aided in reducing the information processing overheads related to manually classifying petabytes of satellite imagery.

It is important to highlight that the BA_FN_ layer aggregations contain buildings along with roads, green area boundaries, footpaths, parking lots, etc. These additional built-up structures are not relevant to the current analysis and can occupy significant BA_FN_ areas in a low-density built environment. Methodological uncertainties can also arise due to the heterogeneity of impervious landscapes and due to misclassification caused by the similar spectral signatures of other built structures on the ground, but at a global ROI, these errors form a very small percentage of the ground truth. This underscores the need to downscale the BA_FN_ layer globally to accurately represent a harmonized rooftop area.

**Fig. 2:**
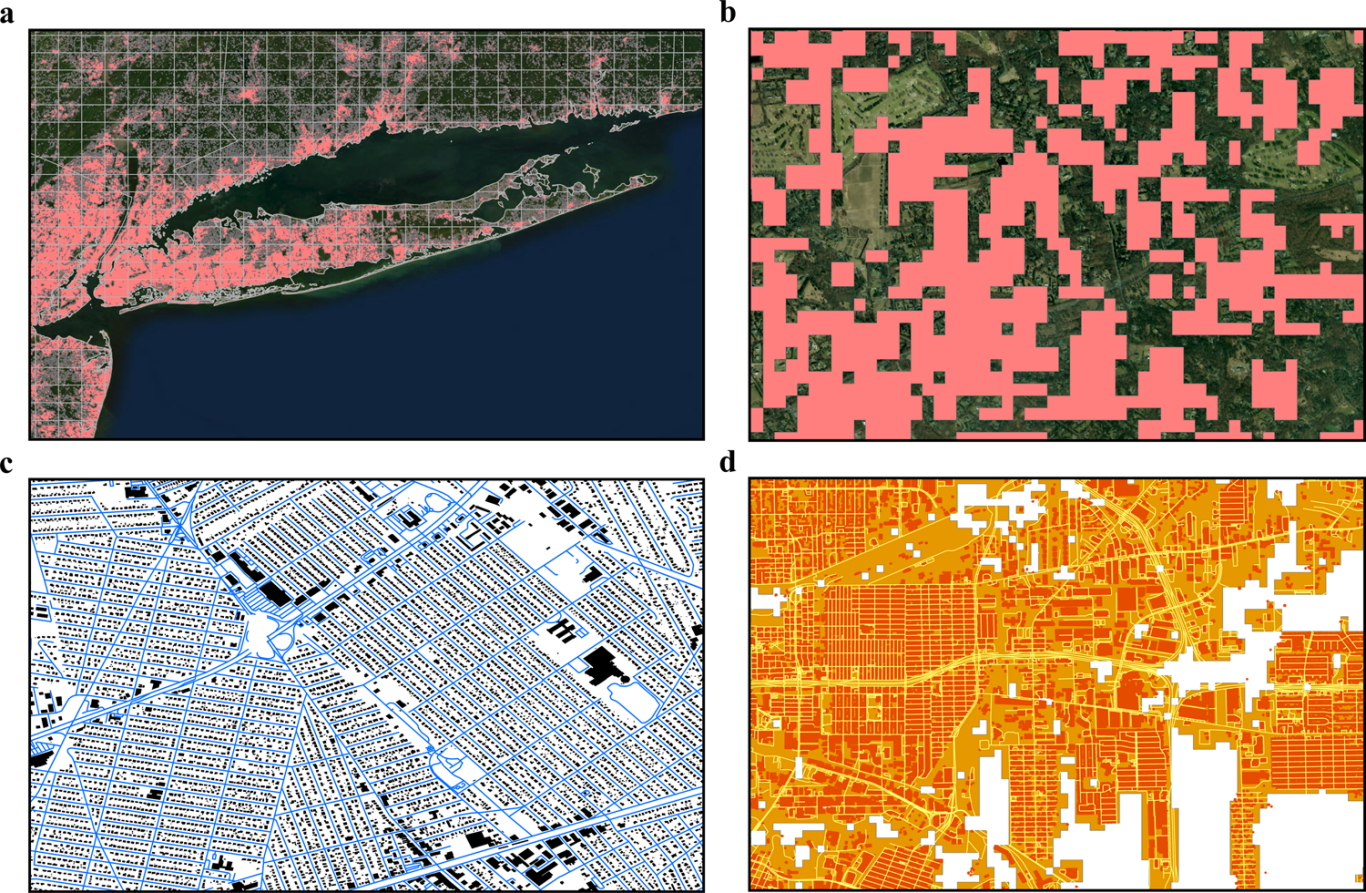
Visual representation of a sample FN involved in Bottom-up methods. **a** The image is depicting an area over New York, USA. The gridlines are the geographical boundaries of Fishnet cells (FN), with each cell size of 10 km^2^. The red area inside each FN is the built area (BA_FN_). **b** The green areas in the image are the non-built-up geography in the year 2015, with red areas representing BA_FN_ as 100 m^2^ blocks. Each block has a value of 0–100% based on the percent of built-up area in a block. **c** The black polygons are the building footprints (BF_FN_) derived from big data sources, inside a sample FN. The blue lines are the roads (RL_FN_) inside the sample FN, with white areas representing empty spaces that are not used in our analysis. **d** The image represents the four main categories of objects present inside each sample FN. The orange areas are the BA_FN_ blocks, with red areas representing the actual buildings, yellow lines representing roads, and white areas are the areas not considered in our study. Data Credits: Copernicus GLC Landcover, Microsoft Building Footprints, and Open Street Maps.

**Fig. 3:**
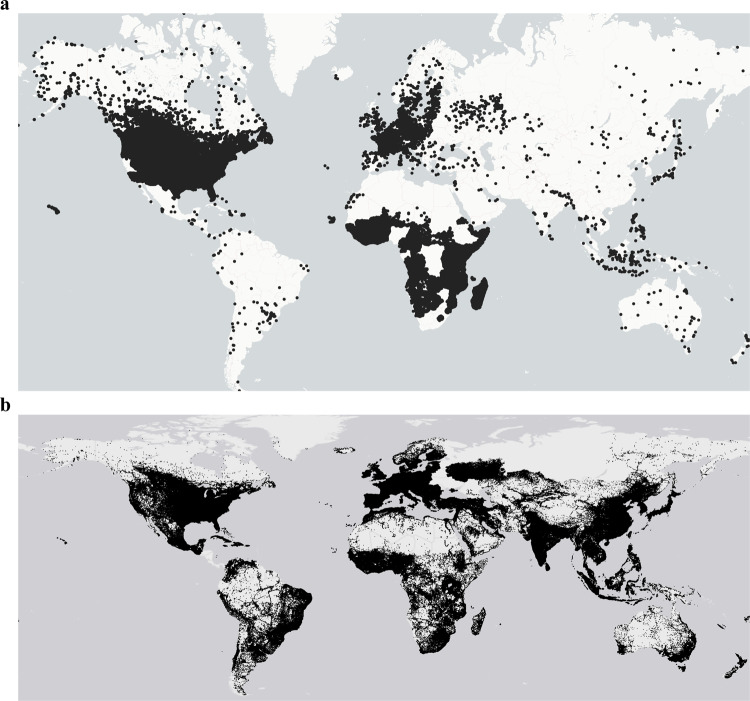
Geographical distribution of global roads and sample Fishnet cells. **a** Global distribution of sample FN was generated in our study. Microsoft AI-based building polygons are used for the USA, Canada, and Tanzania, Ecopia AI-derived building footprint areas are used for the African countries. OSM-derived building polygons representing a total of 4000 sample FNs are used for the rest of the world. **b** Geographic location of all the global roads used in our analysis. OSM-derived global roads have near-global coverage, with each pixel on the map being for each road feature inside the country of reference. Data Credits: Fig. 3b, © OpenStreetMap contributors.

To downscale the BA_FN_ layer, we started by generating a subset of global FN containing actual building footprints^40,42^ (BF_FN_), road length^56^ (RL_FN_), and population count^57^ (PPLN_FN_). For calculating building footprints (derived from Microsoft AI, Ecopia AI, and Open Street Map-OSM datasets), we aggregated individual building footprints based on FN cells overlapping the individual buildings to generate a single building footprint area value per FN (Fig. 2). In total, 37,115 km^2^ of building footprint area and 11.4 million km^2^ of the total land area were covered by our sample FNs, accounting for >300 million individual buildings ranging from small outdoor sheds to mega factories. The building samples also provided us with a diverse set of building types in different geographies spanning a wide spectrum of socio-economic stages of development. These global sample sets (Fig. 3a, Supplementary Table 1) are a marked improvement over the previous literature, where a narrow sampling strategy often at a city/country level is undertaken. Using heterogeneous global building samples enables the overall analysis to be more resistant to generalization error which is introduced due to overreliance on a small set of similar built-up landscapes.

**Fig. 4:**
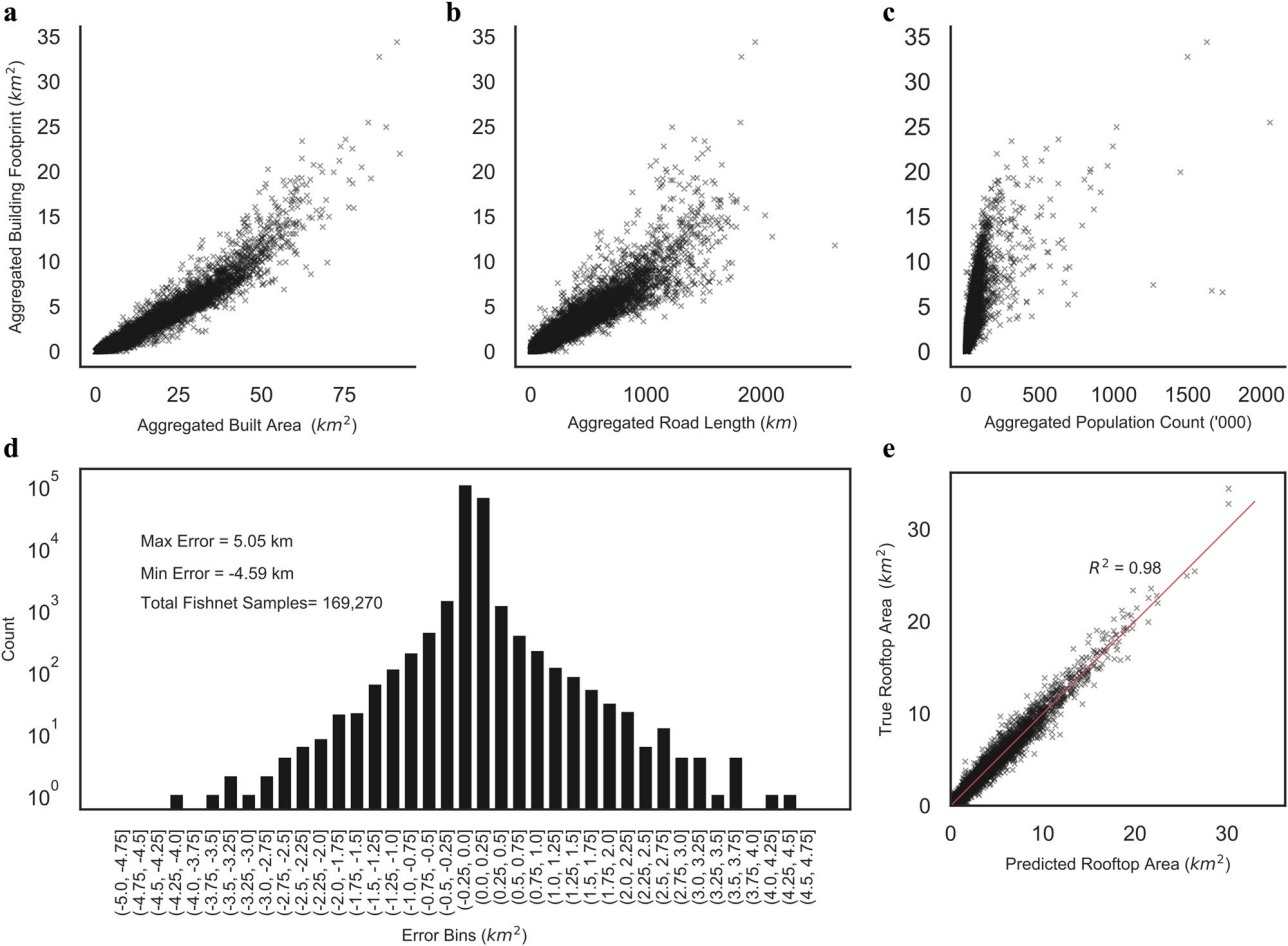
Relationship between inputs, distribution of errors, and prediction accuracy of the downscaling model. The distribution of BA_FN_ (Built Area) (**a**), RL_FN_ (Road Length) (**b**), and PPLN_FN_ (Population) (**c**) in relation to the BF_FN_ (Building Footprint). The distributions have a Pearson Correlation of 0.97, 0.95, 0.72, respectively. The distribution of errors in prediction (**d**) has a bell shape when using the trained model to predict the sample dataset. Each bar is the count of FN that is in each error bin. The majority of errors lie in the −0.25 to +0.25 km^2^ per FN range, with peak error capping at ±5 km^2^ per FN. The downscaling model built upon the machine learning model has shown adequate accuracy in prediction (**e**) with an *R*^2 ^= 0.98. Each cross represents the predicted value of each sample FN. The density of points is higher for low values as the majority of sample FN will have smaller built-up areas contained in them, with very few covering major cities due to the size of the FN used in this study.

OSM provides a highly accurate open-source global roads infrastructure dataset^58^, which we used for mapping road length (RL_FN_). Our sample dataset showed a Pearson Correlation of 0.95 (Fig. 4b) between the building footprint sample and the road length sample. It can be observed that in the real world, the development of roads is often associated with increasing population size and with increasing building stock. Road length has not been used in any previous studies and will increase the accuracy of downscaling over previous methods. In total, we processed >16 million km of roads for our sample FNs (Fig. 3b).

For our population count aggregation, we utilized WorldPop’s high-resolution griddled population count. The high-resolution population dataset coupled with a high-resolution BA_FN_ layer enabled us to accurately map the population count for each of our sample FN. Previous studies at country/regional scale have used coarse resolution (≥1 km resolution) population count which can lead to inaccuracies in downscaling due to over counting of the population in a given FN.

Aggregation and processing of big data like geospatial building and road datasets require a very high computation time and significant cost in hardware. To overcome this challenge, we used Google Earth Engine’s (GEE) cloud computing platform to perform planetary-scale analysis, where the datasets were split into smaller raster files based on each FN’s geographical extent and finally aggregated to represent a single value (see “Methods” section). It should be noted that the GEE platform is lacking in the processing of vector data like building and road polygons, for which we utilized multi-core processing enabled by ArcGIS PRO desktop software. This combination of GEE for raster data (PPLN_FN_, BA_FN_) and ArcGIS PRO for vector data enabled us to process our datasets at a fraction of the time and cost. To our knowledge, this is the first attempt at merging two fundamentally different service platforms to assess global potentials, which could be useful for other planetary-scale resource assessments. Links to the input datasets and their validation reports are documented in Supplementary Table 2.

Next, we linked the aggregated BA_FN_, PPLN_FN_ datasets from Top-down methods and processed BF_FN_, RL_FN_ datasets from the Bottom-up methods using a ML model that takes BA_FN_, PPLN_FN_, RL_FN_ datasets as independent variables and BF_FN_ as the dependent variables. The ML model trains from the relationship between the independent and dependent variables for our sample FNs and estimates the aggregated building rooftop area for the rest of the non-sample FNs, effectively downscaling the BA_FN_ layer to an estimated area occupied by building rooftops (BFE_FN_). For data gaps in PPLN_FN_, RL_FN_ layer, we used an iterative imputing method to interpolate missing values in the dataset using multiple regression runs.

In our tests, the ML model built upon XGboost algorithm provided better accuracy (*R*^2^ value) and better overall error reduction (considerably lower mean absolute error) in the prediction of sample data as compared to multivariate regression (Supplementary Table 3). To reduce the overfitting of the ML model, a 10-fold cross-validation strategy was used for tuning hyper-parameters to generate the best model (see “Methods” section). The ML model performed well (Fig. 4d, e) with ±4 km^2^ error in predicting total building footprint per 100 km^2^ FN, with the majority of errors lying between ±0.25 km^2^. Also, for a total of 37,000 km^2^ of absolute building footprint area across our samples, a total of 4 km^2^ of absolute error was recorded. On a regional level, slight left skewness/systematic underestimation of rooftop area was observed for the Asian Region (Supplementary Fig. 1). At the end of the downscaling step, a dataset containing 3,521,120 FN cells is generated, with each cell containing aggregated building rooftop area (BFE_FN_).

**Fig. 5:**
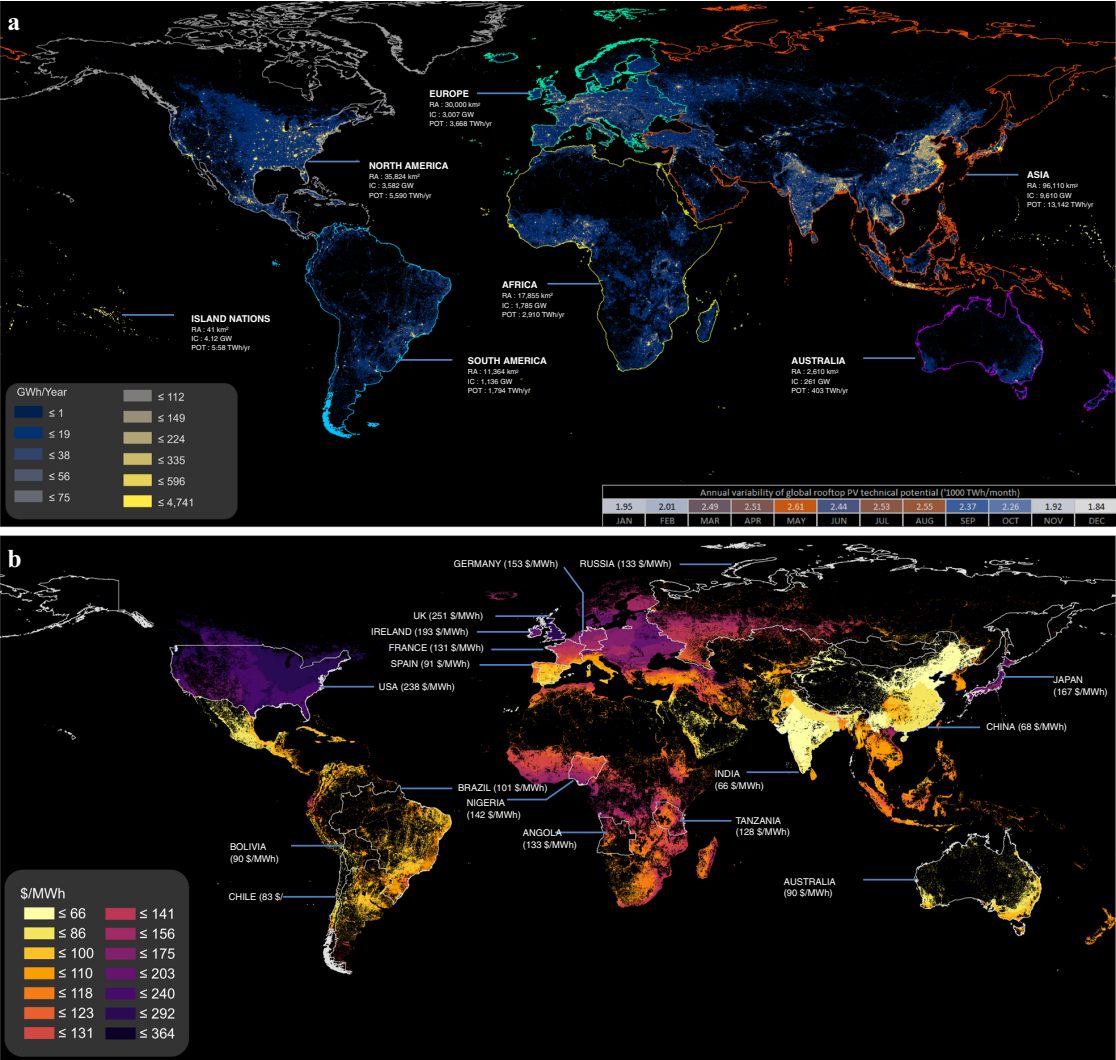
Global Distribution of RTSPV technical potential and LCOE values. **a** The geographical distribution of global RTSPV technical potential generated in our study. Major world regions are highlighted in the image and data is provided for the total estimated rooftop area (RA), installed capacity (IC), and potential generation (POT) for each region. The global landmass is color-coded into 11 RTSPV potential bins. Seasonal variation in aggregated global potential is highlighted in the bottom right. **b** The geographical distribution of LCOE values generated in our study across the globe with major countries highlighted in each world region. Each highlighted country has a corresponding aggregated LCOE value for the whole country. The LCOE values are color-coded into 14 cost bins. Map boundary data from the Database of Global Administrative Areas (GADM: https://gadm.org).

Rooftop area to solar potential conversion was undertaken by using World Bank’s conversion factor^59^ (CF_FN_) for locations between 60°N and 45°S, covering over 99% of the world’s population. The CF_FN_ layer is provided as a griddled raster dataset representing kWh produced by each kWp installed capacity per day. The locations outside the latitudes were assigned a constant CF value of 3.5 kWh/kWp/day (peak). Next, CF_FN_ values for each of the twelve months were used to generate monthly solar potential per FN (SP_FN_,_M_). Further, we aggregated monthly technical potentials to yearly technical potentials for each of the countries to generate a global RTSPV potential map, as shown in Fig. 5. To represent the costs associated with the technical potential, we used the LCOE metric (see “Methods” section). The capital expenditure (CAPEX) values range between 840 and 3874 $ kW^−1^ of installed capacity for 17 countries in total, with the rest of the world being allocated averages for the continent they are in. The operation and maintenance cost (OPEX) and discount rates (DR) were based on whether the country is OECD or Non-OECD. For each FN, LCOE costs were calculated based on CAPEX, OPEX, SP, and DR with a project lifetime of 25 years. Our technology cost data are based on IRENA^2^ residential solar PV costs for 2019.

### Global technical potential and cost assessment

Our assessment shows a total global technical potential of 27 PWh yr^−1^, of which Asia (13 PWh yr^−1^), North America (5.5 PWh yr^−1^) and Europe (3.6 PWh yr^−1^) represent a majority of the potential followed by Africa (2.9 PWh yr^−1^) and South America (1.7 PWh yr^−1^). The hotspots for the potential are concentrated in and around the densely populated nucleated settlements globally (Fig. 5a). Nearly 20% (5 PWh yr^−1^) of the global potential is located within the areas with a high population density (>1500 people/km^2^), with 55% (15 PWh yr^−1^) of the potential being dispersed within the low-density areas (<500 people/km^2^). Amongst the countries, China (4.3 PWh yr^−1^), the USA (4.2 PWh yr^−1^), and India (1.7 PWh yr^−1^) have the highest yearly potential (Table 1). A ±1% deviation can be observed in the yearly global potential due to the aggregation methodology of the CF factor (Supplementary Fig. 2).

**Table 1:** 32 Regions global distribution of rooftop area and solar potentials.

32 World regions^a^	Land area (1000 km^2^)	BA_FN_ (km^2^)	BFE_FN_ (km^2^)	Potential (TWh yr^−1^)^b^
AFE (Africa East)	4355	6921	1246	214
AFN (Africa North)	5485	18631	3398	592
AFS (Africa South)	5537	18132	2443	413
AFW (Africa West)	13130	59604	8613	1317
ANZ (Australia and NZ)	7971	15581	2610	404
ARG	2780	9330	1749	285
ASC (Asia Central)	6622	36053	4981	748
ASE (Asia South East)	2334	62144	9568	1354
ASO (Asia other)	354	13121	2117	304
ASR (rest of Asia)	1642	8942	1561	219
BRA	8533	31962	6386	997
CAN	9904	13851	2394	327
CHN	9380	273585	35156	4375
ENE (Non-EU East)	1035	34634	4111	499
ENW (Non-EU West)	466	4199	630	75
EUE (Europe East)	1138	47700	5457	648
EUW (Europe West)	2984	134286	17467	2210
GBR	244	15798	2400	238
IDN	1899	35710	6163	878
IND	3160	78971	11731	1815
IRN	1623	12437	2004	341
JPN	372	41533	7942	1044
KOR	100	8758	1439	201
LAM (Latin America)	7231	27268	5028	805
MEA (Middle East)	1619	15742	2628	462
MEX	1956	20502	4037	720
ROW (rest of the world)	2426	1302	212	35
RUS	16832	58175	8038	941
SAU	1920	6333	918	169
TUR	780	13526	1716	265
USA	9457	160479	27585	4247
ZAF	1221	11954	2150	374
TOTAL	134491	1297163	193875	27512

^a^ISO alpha-3 codes are the region names for individual countries, a group of countries as a region has names described in parenthesis.

^b^Considering 100% rooftop availability at 10% panel efficiency.

Although the African region has a good solar insolation endowment, the RTSPV potential is assessed as being the third lowest due to low building stock. Amongst the African Region, the largest potential is concentrated in the West African region followed by the North African Region. The combined West and North African regions have more potential than India, highlighting the importance that low-cost RSTPV can play in future energy systems. Future population growth and a corresponding increase in the building stock may increase the overall RTSPV potential for Africa. Both North American and European regions have similar assessed rooftop areas (~30,000 km^2^), yet North America has nearly 1.5 times the potential of Europe due to higher solar insolation during the year especially during the winter months.

**Fig. 6:**
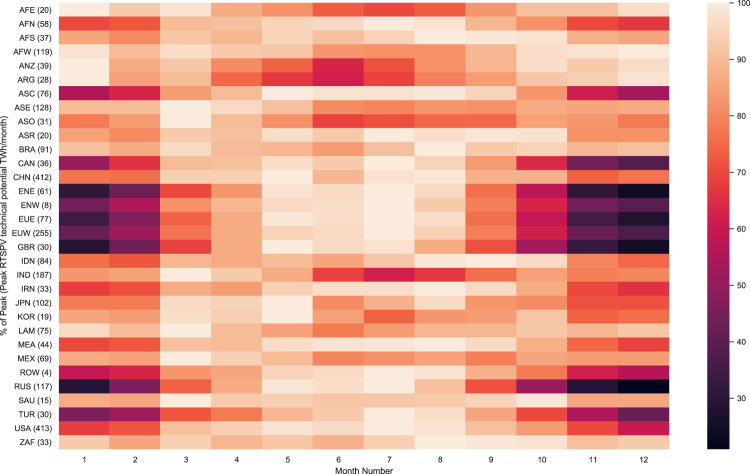
Monthly potential variability for 32 world regions. The heatmap represents the monthly variability of regional potentials. The values in the parenthesis represent the highest monthly potential for the region. The color-coded heatmap represents % of the maximum monthly potential of the region. Europe (EUW, EUE) has shown high intra-annual variability of the potential, with the maximum potential being realized in the summer months. In the southern hemisphere, the maximum potential is realized in the winter months. Africa West (AFW) has shown the lowest variability in the monthly potential.

Along with spatial variability due to on-ground building distributions, seasonal variability of the RTSPV potential is also observed due to the variation in intra annual solar insolation. The seasonal variability of the monthly global potential is between 1.84 and 2.61 PWh, with December and January representing the months with the lowest global potential (Fig. 5a). Globally the highest seasonal variability of the potentials is observed above the 45° north latitude covering Europe, Russia, the USA, and Canada. Within regions, the highest intra annual variability of the potentials (Fig. 6) is observed in the West European region (EUW) with monthly potentials between 94 and 255 TWh. There is a variability of ±40% around the average monthly potential of 183 TWh in EUW, with the highest monthly potential being observed in the summer and the lowest monthly potentials in the winter. The lowest regional intra annual variability of potentials is observed in the West African region (AFW) with monthly potentials between 97 and 119 PWh. There is a variability of ±1% around the average monthly potential of 109 TWh in the AFW region, with the maximum monthly potential observed in December and January.

**Fig. 7:**
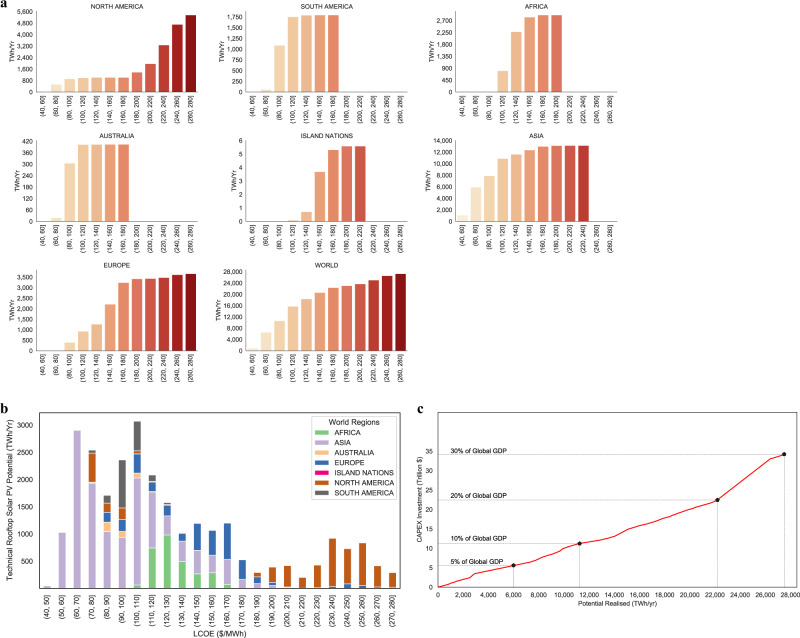
Regional supply cost curves based on a 20 $/MWh bin size and distribution of LCOE across regions. Regional Supply cost curves (**a**), showing the cumulative technical potential for a given LCOE bin size. The absolute increase in the bar height represents the change in the added potential that can be realized in a given LCOE bin. Near constant bar heights for consecutive bins are representative of a very small additional potential being realized at an increasing cost. For a single bar, the height of the bar represents the cumulative potential that can be realized within a specific LCOE band. **b** Distribution of potentials across LCOE bins of 10 $/MWh size. Potential extraction is the cheapest in Asia, followed by Africa and Europe. **c** CAPEX investment required to achieve a specific total potential as a percentage of global GDP in 2015. Nearly 50% of global potential can be achieved at investment equivalent to 15% of 2015 global GDP value.

To analyze the cost of attaining the potentials, we generated supply cost curves for seven world regions and also at an aggregated global level (Fig. 7a). Nearly 10 PWh yr^−1^ (40%) of the global potential can be achieved below 100 $ MWh yr^−1^, with the majority of the potential being realized below 200 $ MWh^−1^ (Fig. 7b). At a global level, nearly 40% of the potential can be achieved with an investment equivalent to 10% of the 2015 global GDP value, and with an investment equivalent to 30% of the 2015 GDP value, nearly 100% of the global potential can be realized (Fig. 7c). We found that realizable potential doubles with each subsequent doubling of capital investment in RTSPV until invested capital is equivalent to 20% of the global GDP value in 2015. An increase of investment from 20 to 30% of the global 2015 GDP value increases the realizable potential by only 27% indicating the areas where the cost of implementation of RSTV is very high. These areas are represented by large warehouse/industrial complexes in Alaska and Canada, where yearly solar insolation is low and CAPEX investment is high to cover the entire large rooftops with solar panels.

At a global level, spatial variability in the LCOE is also observed (Fig. 5b). In the northern hemisphere, the LCOE values gradually increase from 40 $ MWh^−1^ to 280 $ MWh^−1^ with increasing latitude. Here North-East China is an exception which has shown a decrease in LCOE values with increasing latitudes. For Asia, a majority of the potential can be realized between 40 and 100 $ MWh^−1^ making the RTSPV competitive with fossil fuel technologies. The cost of attaining the country-specific potential is lowest in India at 66 $ MWh^−1^ compared to China (68 $ MWh^−1^).

For Europe, Africa, South America, and Island Nations a majority of total potential can be realized below 180 $ MWh^−1^. Within Europe, Spain has the lowest LCOE cost of 90 $ MWh^−1^, with an increasing trend in cost being observed when moving towards the higher latitudes. Within each country of the European region, further variability in LCOE is also observed with some regions observing cheaper costs than neighboring regions in the same latitude. In the African region, the majority of the total potential can be realized between 110 and 160 $ MWh^−1^. Within Africa, Nigeria, Gabon, and Cameroon have the highest costs (around 150 $ MWh^−1^) to achieve their respective potentials.

North America, UK, and Japan have shown the highest cost to realize the potential. This can be attributed to the high CAPEX costs in the countries, which are expected to reduce in the future due to technological innovations and reduction in import tariffs. In North America, Canada and the northeastern states of the U.S.A around the Great Lakes have the highest LCOE costs. The cost to achieve the potential in these countries ranges from 200 to 280 $ MWh^−1^. U.K (251 $ MWh^−1^) has the highest country-specific costs to achieve its potential.

**Fig. 8:**
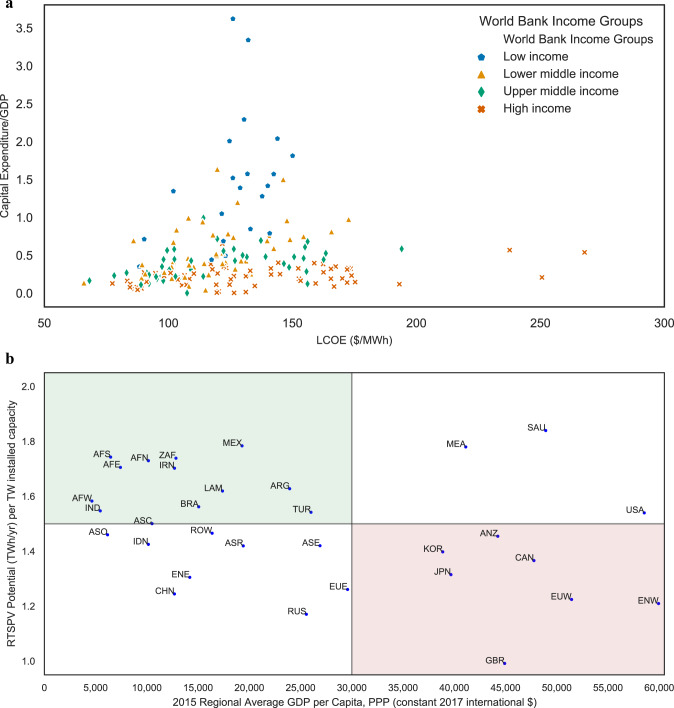
Global economic and technical variation in accessing the potentials. **a** Disparity in achieving full potential, with low-income countries requiring capital investment greater than their respective 2015 GDP value to achieve full potential. **b** Distribution of 32 regions in relation to their relative yield factor and GDP per capita. The red region focuses on countries that currently have high rooftop PV rollout. The green region focuses on countries where RTSPV technology rollout will have maximum benefits.

Globally, CAPEX required to access potential varies both with respect to the size of the GDP and with respect to the LCOE values (Fig. 8a). To realize the complete potential in their respective countries, low-income countries would need to invest capital which is multiple times (up to 3.5 times) their 2015 GDP value even at relatively low LCOE of between 80 and 150 $ MWh^−1^ to cover for high upfront costs. For similar LCOE values, high-income countries (World Bank Income Classification) can achieve their complete potentials with a capital investment equivalent to a fraction (up to half) of their 2015 GDP value.

We categorized countries into groups based on their GDP per capita (GDPC) and on their yield factor (Fig. 8b). In this study, we defined the yield factor as the yearly potential that can be realized from 1 TW of installed capacity. Based on this categorization, we found out that emerging economics including India, Brazil, and Mexico have high yield factors (1.5–2) that would favor the deployment of RTSPV in these countries. Even though the LCOE difference between the two most populous countries (India and China) is minimal, with the greater solar endowment and with high yield factor, RTSPV technology rollout becomes more favorable in India compared to China. However, the uptake of RTSPV is still very low in these countries due to lack of credit and the inability to pay the high upfront cost of the RTSPV system. This highlights the need for global cooperation, technology transfer, and green financial instruments to accelerate the deployment of low carbon RTSPV technology in low-income and medium-income countries.

## Discussion

The study showcases how a framework based on big data and an ML model in conjunction with cloud computing platforms can be used to undertake a planetary scale resource potential assessment. We analyzed 130 million km^2^ of global land surface area by utilizing learnings from global samples containing 300 million buildings with 16 million km of roads. Using Google Earth Engine and a ML model we demarcated 1.2 million km^2^ of the built-up area containing 0.2 million km^2^ of rooftop area. As part of the assessment, we generated (1) a global rooftop area dataset (2) a global RTSPV potential dataset at a monthly temporal resolution, (3) costs of attaining the technical potential. The datasets were further used to generate high-resolution global maps of the potential and costs. We have also advanced the current state of art by combining the top-down and bottom-up approaches at a global scale to develop a hybrid framework for resource potential assessment which can also be used in advancing the assessment of global wind and bioenergy potentials. The assessment shows that a sizeable RTSPV potential of 27 PWh yr^−1^ exists at a global level that can be attained for costs between 40 and 280 $ MWh^−1^. The potential is highest in Asia followed by North America and Europe. A capital investment of around 7 trillion dollars is required at current prices to achieve a global RTSPV based electricity generation of 10 PWh yr^−1^ below the LCOE of 100 $ MWh^−1^, covering 3.72 billion people globally.

At the EU-27 regional level, our estimated rooftop area of 7596.4 km^2^ is similar to the 7935 km^2^ calculated in the Bodis et al. study when incorporating a rooftop scaling factor of 0.3. For the USA, our estimated rooftop area and annual potential of 8827 km^2^/1.9 PWh yr^−1^ compare well with the estimates of 8130 km^2^/1.4 PWh yr^−1^ presented in the Gagnon et al. study when incorporating a rooftop scaling factor of 0.32. On a city level basis, our potential of 1 TWh yr^−1^ is in alignment with the 1 TWh yr^−1^ calculated in a study by Hong et al.^36^ where they have used advanced hillshade analysis to capture the effects of building induced shadows in a dense urban topography. A detailed global/regional/country and city level comparison of our results with selected research work is documented in Supplementary Table 7. From the comparison with other studies, we can conclude that the results from our framework demonstrate high veracity as they are within the margin of error of values present in the literature. In addition, the good estimation accuracy of our framework at high spatial resolution feeds into higher accuracies at aggregated lower resolutions.

Our assessment has important implications for addressing the twin challenges of sustainable development and climate change with co-benefits in advancing SDG 3 and SDG 7. First, the analysis of spatial RTSPV potential presented in this study shows that 55% of the global RSTPV potential is spread across low-density areas. This highlights an important aspect of solar transition, where the majority of its benefits in providing cost-effective and fast deployable electricity can be realized in rural areas. RTSPV can thus aid in mitigating the energy poverty being experienced in the less developed and sparsely populated areas in a country where extensive grid integration may be costly or where competition for land may exist. Nearly 20% of the global potential lies in the high-density areas where the deployment of RTSPV can aid in displacing fossil fuel-derived electricity with less polluting electricity generation thereby reducing local air pollution^60^. Second, from a perspective of energy equality and “leaving no one behind” agenda of the SDG, the most disadvantaged areas with respect to access to electricity are currently the low-income countries^3^ which require the rapid and cost-effective deployment of clean electricity generation infrastructure. Our assessment shows that low-income countries may need significant capital investments as steep upfront RTSPV installation costs in the order of magnitude 2–3 times their 2015 GDP value to achieve their country-specific potentials. At current costs, governments may need to provide subsidies and seek external investments to improve the prospects of deployment of RTSPV in these areas. This highlights the vital role that the developed economies may play in enabling the deployment of RTSPV in these countries by the means of financial flows to realize the climate change co-benefits. With maturing of the technology and the emergence of an economy of scale, costs will further go down to enable a solar revolution in these areas and aid in their low carbon energy future.

Third, countries that are currently reaping their demographic dividends like India and China are better suited for rapid deployment of RTSPV. We showed that these countries have high potential with low seasonal potential variability along with the low cost of deployment. As these countries have the largest population share globally with large building stocks, they can be the first movers in decarbonizing their electricity generation infrastructure by substantially deploying a decentralized electricity generation portfolio, further aiding in climate change mitigation. Along with climate mitigation, RSTPV deployment in these countries can gain a lot from the high workforce percentage in the population in the form of cost-effective manufacturing and operational maintenance. Fourth, the high-resolution assessment can aid the local governments in identifying suitable locations for the rapid deployment of energy generation infrastructure. This way bottom-up formulation of energy policy can lead to inclusive designing of nationwide policies to provide energy justice to the citizens. Fifth, businesses and financial institutions like World Bank and International Monetary Fund can analyze in-depth the investment opportunities and risks in implementing RSTPV infrastructures leading to local job creation and sustainable development of manufacturing industries. Sixth, the information contained in the assessment along with supply cost curves is a measurable step forward and fills in a significant information gap that is present in current integrated assessment models where solar PV potentials are often represented as aggregated potentials for utility and rooftop installations. Our assessment provides insightful findings and technical potential datasets that will certainly aid in accurately modeling the future carbon-neutral scenarios to inform national energy policies^61–66^. This will no doubt aid in exploring sustainable and inclusive low-carbon future possibilities.

Our study shows pronounced variability of seasonal potentials in the higher latitudes for countries covering Europe, North America, and Australian regions. These regions have high electricity consumption per capita along with the financial capacity to introduce significant VRE in the electricity generation mix. To mitigate the variability of the potentials over the year, smart grids that optimize the generation portfolio and the introduction of regulator-driven mechanisms that balance the generation market becomes important. Further, the introduction of new market mechanisms^67^ is needed to effectively integrate the prosumers and utility operators in competitive electricity generation markets. With falling prices of electricity storage technologies and smart management of interconnected grids, RSTPV technology will play a critical role in these markets by undertaking generation, storage, and system balancing roles.

In conclusion, our assessment shows that the current electricity generation potential of RTSPV exceeds the current (2018) yearly aggregated global electricity demand^68^. Our assessment also shows that a minimum of 50% of the total global rooftop area is required to meet the yearly global aggregated electricity demand. Due to the diurnal cycles of solar insolation and to balance the seasonal and daily variability of the RTSPV generation, the role of storage solutions to compliment RTSPV electricity generation is critical in realizing the maximum potential of this technology and to meet the peak daily demand. Hence, the practical realizable potential of RTSPV will depend on the future cost trajectory of storage technologies, capital expenditure related to the technology, and the overall configuration of the energy system.

Even with its limitations and shortcomings the current assessment is still the state of art and provides researchers with global analysis datasets. The underlying methods and datasets have been peer-reviewed and are of the highest quality currently available and represent a generation advancement over the datasets used in the current state of art methods. The current dataset can be improved by using a next-generation 10 m resolution landcover dataset and with an increase in the spatial resolution of the population and solar data at a global scale. Better data processing platforms can enable further work to be accomplished at a 1 km resolution providing a 100 times increase in the spatial representation of the potentials. In addition, inputs in the form of realistic regional variation in the rooftop availability will aid in narrowing down the uncertainty in potentials and cost and should be the next logical research step in model improvement.

## Methods

### Top–down method

We generated a total of 3,521,120 fishnets using the ArcGIS PRO desktop application for all the global landmass except for the continent of Antarctica. The FN grid is the lowest unit of data aggregation in our method. The FN’s at the boundary of two countries have a common Fishnet identifier but unique country attribution with FN being split at the boundary. Next, the fishnet polygons were uploaded to Google Earth engine platform^69^ (GEE) to calculate satellite-derived Built Area (BA_FN_), Population (PPLN_FN_), and conversion factors (CF_FN_) for each fishnet cell.

We utilized the global land cover (LC) layer from Copernicus Global Land Service v2.0 to calculate BA_FN_ values within each FN. This landcover classification layer was chosen for its robustness and near-global coverage and it is backed by exhaustive testing and validation. The LC has many categories of classifications, amongst which built-up area is one of the classifications. The built-up classification is in turn derived from European Space Agency’s World Settlement Footprint 2015 layer that is derived from a 10 m resolution sentinel mission’s radar and optical imagery. To calculate the total built-up area in each Fishnet cell, the LC raster file was first cut into smaller sizes based on each fishnet’s geographical bounds. The individually cut LC dataset was then aggregated using the following:

1 $${{{{{{\mathrm{BA}}}}}}}_{{{{{{\mathrm{FN}}}}}}}={\sum }^{}({{{{{\mathrm{P}}}}}}{{{{{{\mathrm{X}}}}}}}_{{{{{{\mathrm{V}}}}}}}\times {{{{{\mathrm{P}}}}}}{{{{{{\mathrm{X}}}}}}}_{{{{{{\mathrm{A}}}}}}})$$

where BA_FN_ is the Built-up area in each fishnet cell, PX_V_ is the pixel value (0–100) representing the percent of the built area in each pixel, PX_A_ is the area occupied by each pixel.

To map the number of people living in each fishnet cell, we utilized a global population raster file at 100 m resolution provided by the WorldPop project. The population raster disaggregates the recorded United Nations population counts in an administrative unit to a finer resolution using ML-based methodology. The population raster file was split into smaller entities based on each fishnet cell’s geographical bounds. Then, a masking file containing areas covered by the LC layer within each fishnet cell is used to mask the population outside the bounds of the BA_FN_ area in each fishnet. The individual small population dataset was aggregated for each fishnet cell using the following:

2 $${{{{{{\mathrm{PPLN}}}}}}}_{{{{{{\mathrm{FN}}}}}}}={\sum }^{}{{{{{\mathrm{PX}}}}}}{({{{{{\mathrm{NM}}}}}})}_{{{{{{\mathrm{V}}}}}}}$$

where, PPLN_FN_ is the total count of people living in each fishnet, and PX(NM)_V_ is the pixel value of each pixel that is not masked by the overlay LC layer. The population raster file was masked to remove any population count data that is not part of the LC pixel. The masked population raster pixel can be attributed to artifacts induced due to the downscaling algorithm in the original dataset or extra built-up areas that were external to the LC layer. To maintain homogeneity in the analysis, the LC layer was taken as the base for all analysis, and any area not covered by (BA_FN_) was assumed to be not present on the ground, even if it actually exists as ground truth.

The CF factors were calculated using World Bank’s SolarGIS raster datasets. The dataset is provided as a 1 km resolution raster dataset with each pixel providing daily kWh generation for each kWp (peak) installed capacity within that pixel at a monthly resolution. The dataset has been generated using extensive simulation and validation of solar insolation, power conversion losses, effects of atmosphere, and panel aging using 20 years of documented data. For each fishnet cell, the CF factors were aggregated using the following:

3 $${{{{{{\mathrm{CF}}}}}}}_{{{M}},{{{{{\mathrm{FN}}}}}}}=\frac{1}{n}\mathop{\sum }\limits_{1}^{n}{{{{{\mathrm{P}}}}}}{{{{{{\mathrm{X}}}}}}}_{{{{{{\mathrm{V}}}}}}}$$

where, CF_*M*,FN_ is the CF factor for each month for each FN, *n* is the number of CF pixels in the FN and PX_V_ is the pixel value of each pixel in the FN geographical bound. All three datasets (PPLN_FN_, CF_FN_, BA_FN_) and FN geometries were first processed on the ArcGIS Pro desktop application to harmonize coordinate reference systems and then uploaded to the GEE platform for processing. On GEE, we split the datasets based on individual FN geometries and aggregated the three datasets based on the rules highlighted above. GEE’s cloud computing architecture can process a large number of datasets in a relatively shorter time frame and outputs a tabulated dataset for further processing.

### Bottom-up method

To generate ground truth building footprints, we collected building polygon shapes as vector layers from big data sources. For building footprint samples, we used AI-generated building footprints by Microsoft AI and Ecopia AI teams. These two datasets cover the entire USA, Canada, and 39 African countries. The samples from Microsoft AI and Ecopia AI (>300 million individual buildings) were split up based on the FN layer for each FN cell overlapping the sample countries, further masked to remove building footprints outside of the BA_FN_ layer. The unmasked building footprints were aggregated based on the following:

4 $${{{{{{\mathrm{BF}}}}}}}_{{{{{{\mathrm{FN}}}}}}}={\sum }^{}{{{{{\mathrm{BP}}}}}}{({{{{{\mathrm{NM}}}}}})}_{{{{{{\mathrm{V}}}}}}}$$

where BF_FN_ is the aggregated building footprint for each sample FN and BP(NM)_V_ is the unmasked individual building footprint polygon area within the FN that is overlapping the BA_FN_ layer. The masking removed buildings constructed after the 2015 reference year. Although the BA_FN_ layer covers the entire extent of the built-up area globally, it can still miss some built-up areas due to artifacts in satellite imagery. However, this is negligible and for the purpose of our study considered as the reference layer on which to base our analysis. Overlapping building footprints were dissolved into a single polygon before splitting and polygons being intersected by an FN boundary were split up at the line of intersection. For the rest of the world, OSM-derived building footprints (accessed April 2020) were analyzed and aggregated based on Eq. (4). A total of 4000 global FN samples were selected from the OSM building dataset. The sampling strategy to generate the 4000 OSM samples was to extract buildings bound by FNs that had BF_FN_/BA_FN_ ratio of between 0.15 and 0.11. These ratios correspond to the 75th percentile and 50th percentile of data processed from Microsoft AI and Ecopia AI datasets. In total, we were able to successfully collect samples from nearly all the global countries covering different stages of socio-economic development, cultural spread, and geographical locations.

The road length metric was derived entirely from the OSM datasets. To process the RL dataset, OSM’s planetary dataset file (accessed April 2020) was used. The planetary file has roads represented in the form of lines attributed by the different types e.g., residential, highway, footpath, etc. The line feature was split up based on each global FN, masked using BA_FN_ layer, and aggregated based on the following:

5 $${{{{{{\mathrm{RL}}}}}}}_{{{{{{\mathrm{FN}}}}}}}={\sum }^{}{{{{{\mathrm{L}}}}}}{({{{{{\mathrm{NM}}}}}})}_{{{{{{\mathrm{V}}}}}}}$$

where RL_FN_ is the aggregated road length for each global FN and L(NM)_V_ is the individual length of all the roads within each FN overlapping BA_FN_ layer. It was observed during the aggregation of the RL_FN_ dataset, that road endpoints in the OSM dataset overlap in some locations, these overlaps were dissolved into single line features prior to aggregation. Also, some roads extend beyond the BA_FN_ layer within each FN. These roads were clipped at the BA_FN_ boundary to maintain a homogenous extent for the region of interest for all the datasets used in the analysis. In total, we were successful in processing the road infrastructure for nearly all the countries of the world.

Loading, splitting, and geometry processing for the building footprints and the road lines were performed using ArcGIS PRO’s multicore support. The aggregation of the datasets and mapping of the aggregated dataset with FN boundaries was performed using custom python scripts built on DASK^70^ parallel compute module. Due to the sheer size of the data being processed, we found that multicore architectures and parallel computing frameworks developed in recent years can be of great use in designing and executing planetary-scale analysis with minimal cost and time requirements.

We have used cloud computation in the form of the Google Earth Engine platform. In general, parallelization of the data processing was performed in four ways, first when disaggregating global raster datasets and mapping the aggregated values to our global FNs. We did this task for 12 monthly solar rasters, 1 Landcover raster, and finally for the global population raster. Here, we ran all the raster algebra and mapping codes on Google Earth Engine where the backend data processing was split into multi parallel streams and executed on google cloud infrastructure. This led to the speeding up of the data crunching as raster algebra is highly parallel in its execution where each raster cell can be worked on independently. Second, when disaggregating global vector datasets and mapping the aggregated values to our global FNs. As of the time of writing this article, Google Earth Engine was less efficient in undertaking vector analysis. The reason for this is that instead of pixel raster surface which can be computed in a parallel fashion, vector datasets need specialized algorithms. Here, we utilized the multi-core parallel processing architecture of ARCGIS Pro where vector datasets in the form of building footprints and roads can be effectively processed by splitting the map into different regions and processing each region on a single core. This task is performed in the backend using apache spark. Third, when doing data aggregation and manipulation inside desktop python environment. Large components of the data processing ranging from calculating rooftops to LCOE calculations were performed using the DASK framework. In this framework, data frames can be broken down into small chunks and processed in parallel. This provided us with a significant reduction in processing times. Fourth, when training ML model. Custom scripts were written on python to perform hyperparameter optimization and 10-fold cross-validation in a parallel fashion. Each of the 10-folds of the cross-validation was assigned to the independent core of our 12-core machine which led to faster convergence of the hyperparameters to an optimal solution.

### Downscaling model

The ML model was trained on PPLN_FN_, RL_FN_, BA_FN_ as independent variables and BF_FN_ as the dependent variable for each sample FN. The first step in model preparation was to impute the missing data in the independent variables. The imputations are necessary as some FNs have either missing population or road length data due to the global scale of the analysis. This discrepancy is expected and is present due to OSM roads not being mapped for every road on the planet and also due to the downscaling methodology used in generating the original population raster by WorldPop.

Data imputation was handled using a custom python script utilizing the Scikit-Learn^71^ module’s iterative impute function. Further, we generated the downscaling model using XGBoost^72^ framework. When choosing between Neural Network-based framework or Gradient boosting frameworks, we used the latter as XGBoost framework has shown superior performance, while using considerably less computation time to reach an optimum model state. Also, being run on CPU-only architecture, the XGBoost framework can generate repeatable results in each subsequent run, which is difficult to achieve on a GPU-based framework like Neural Networks due to inherent uncertainty induced by the massive parallel compute architecture of a GPU.

The base XGBoost model was customized for our task by hyper-tuning the parameters of the model using 5-fold cross-validation. Each fold of the cross-validation generated a mean square error (MSE) loss metric at the end of its run. The mean of all five MSE was chosen as the metric to reduce during the hyper-tuning process (final model parameters are present in Supplementary Table 1). The trained model was then used to estimate building footprint values for each global FN cell using PPLN_FN_, RL_FN_, BA_FN_ values. The final output of the downscaling (BFE_FN_) was stored as a global 10 km resolution raster file where each pixel represents the estimated aggregated building footprint for each FN.

### Technical potential estimation

To calculate RTSPV potential from BFE_FN_, we made some generalizing assumptions to maintain uniformity in our calculations. We assumed that the estimated building footprint is representative of the available rooftop area in each FN i.e., 100% of the estimated rooftop is available for solar panel installation. To install 1 kWp of roof-mounted solar PV, 10 m^2^ of rooftop area is required, which is in line with the thin film technology currently in use. The roof-mounted solar PV is installed at the optimum angle for each latitude and is sun-facing and shade-free to generate maximum electricity output. The building rooftops are flat in design leading to the utilization of the entire rooftop for the installation of solar panels.

Based on the assumptions i.e., 10 m^2^ area for a 10% efficient panel, the technical solar potential is calculated for all the global FNs for 12 months using the following:

6 $${{{{{{\mathrm{SP}}}}}}}_{{{M}},{{{{{\mathrm{FN}}}}}}}={{{{{\mathrm{BF}}}}}}{{{{{{\mathrm{E}}}}}}}_{{{{{{\mathrm{FN}}}}}}}\times {{{{{\mathrm{C}}}}}}{{{{{{\mathrm{F}}}}}}}_{{{M}},{{{{{\mathrm{FN}}}}}}}\times \frac{{{{{{\mathrm{Day}}}}}}{{{{{{\mathrm{s}}}}}}}_{{{M}}}}{10}$$

where SP is the technical solar potential, BFE_FN_ is the estimated roof area in m^2^, CF is the conversion factor, *M* is the month and FN is the unique fishnet cell, and Days_M_ is the number of days in the respective month. The World Bank’s solar conversion factors are available for regions between 60°N and 45°S. For regions beyond 60°N and 45°S, we assumed a constant conversion factor of 3.5 kWh/kWp/day. This has led to a slight over-assessment of the potential for countries like Sweden, Norway. However, as the density of built-up area reduces significantly beyond the 60°N and 45°S, the total global error due to this assumption remains small. The calculations for generating the solar potential are processed using custom python scripts using the DASK module to handle massive arithmetic operations. Further, the processed solar potential raster dataset is stored as a geopackage file for visualizations and economic calculation.

### Cost calculation

LCOE provides an easy and robust method to compare the economic viability of a project within a specific FN. It was assumed that the capital cost of the installation (CAPEX) will be staggered to the first year of commissioning and the installed panels will have a lifetime of 25 years. The geo-mapping for CAPEX, operating expenditure (OPEX), and discount rate (DR) was sourced from IRENA’s renewable energy cost report 2019. The LCOE for each FN is calculated using the following:

7 $${{{{{{\mathrm{LCOE}}}}}}}_{{{{{{\mathrm{FN}}}}}}}=\frac{\frac{{\sum }_{t=1}^{25}{{{{{\mathrm{CAPE}}}}}}{{{{{{\mathrm{X}}}}}}}_{{{{{{\mathrm{FN}}}}}}}\,+\,{{{{{\mathrm{OPE}}}}}}{{{{{{\mathrm{X}}}}}}}_{{{{{{\mathrm{FN}}}}}},t}}{{(1\,+\,{{{{{\mathrm{DR}}}}}})}^{t}}}{\frac{{\sum }_{t=1}^{25}{\sum }_{M=1}^{12}{{{{{\mathrm{S}}}}}}{{{{{{\mathrm{P}}}}}}}_{{{M}},{{{{{\mathrm{FN}}}}}}}}{{(1\,+\,{{{{{\mathrm{DR}}}}}})}^{t}}}$$

where, CAPEX_FN_ (2019 $/kW) is the capital expenditure in installing the RTSPV system for the given FN, OPEX_FN,*t*_ (2019 $/kW) is the operational and maintenance expenditure for the given FN and for the specific year (*t*), *t* is the year number, DR is the discount rate, *M* is the month number, and SP_*M*,FN_ (kWh/month) is the potential generation for the given month and FN. We have used CAPEX data for 17 different countries and allocated the average CAPEX value to the rest of the countries based on the continent they are in. OPEX and DR have values based on OECD and Non-OECD country classifications. Country-wise aggregation of LCOE, SP from each FN from their respective high-resolution intra country values has been done using the following rule

8 $${{{{{{\mathrm{LCOE}}}}}}}_{{{{{{\mathrm{country}}}}}}}:\left\{\begin{array}{c}{{{{{\mathrm{CAPEX}}}}}}:{{{{{\mathrm{aggregation}}}}}}\_{{{{{\mathrm{of}}}}}}\_{{{{{\mathrm{individual}}}}}}\_{{{{{\mathrm{FN}}}}}}\_{{{{{\mathrm{values}}}}}}\\ {{{{{\mathrm{OPEX}}}}}}:{{{{{\mathrm{aggregation}}}}}}\_{{{{{\mathrm{of}}}}}}\_{{{{{\mathrm{individual}}}}}}\_{{{{{\mathrm{FN}}}}}}\_{{{{{\mathrm{values}}}}}}\\ {{{{{\mathrm{SP}}}}}}:{{{{{\mathrm{aggregation}}}}}}\_{{{{{\mathrm{of}}}}}}\_{{{{{\mathrm{individual}}}}}}\_{{{{{\mathrm{FN}}}}}}\_{{{{{\mathrm{values}}}}}}\\ {{{{{\mathrm{DR}}}}}}:{{{{{\mathrm{mean}}}}}}\_{{{{{\mathrm{of}}}}}}\_{{{{{\mathrm{indiviual}}}}}}\_{{{{{\mathrm{FN}}}}}}\_{{{{{\mathrm{values}}}}}}\end{array}\right\}.$$

It should be noted that we did not consider the cost of additional grid expansion or storage infrastructure to attain the full technical rooftop solar PV potential. Also, the cost of decommissioning and scrap metal value of the installation was not considered at the end of the 25-year lifetime of the projects. While calculating the SP and LCOE, it was assumed that no rooftop solar PV installation exists globally, and all the additional capacities will start their commissioning from the year 2019.

### Limitations

Our assessment is based on the accuracy of the global landcover layer, which with its 100 m resolution can in some locations overestimate the built-up area extents. In addition, the landcover classifies roads, parking lots, boundaries of green areas, tennis courts, and archeologically significant areas as built-up areas with misclassification varying between different regions. Our assumption that the rooftops being flat, shadow-free and sun-facing with a full rooftop available for installation adds to the methodological limitations. Next, the big data related to building footprints and global roads have inherent methodological limitations like the simplified representation of a complex rooftop with a square polygon, overlapping roads, etc.

For technical potential calculations, we assumed that 100% of the estimated rooftop is available for installing solar panels i.e., orientation and slope of the building are not accounted for the 100% rooftop availability assumption-based results in our main analysis. These assumptions can lead to limitations in the real-world interpretation of main results as a fraction of rooftop may be available for the installation of solar panels. To account for this, we have documented regional change in potential as an uncertainty analysis for a combination of rooftop scaling factors and panel efficiencies. In the current literature, reduction of total rooftop area to available rooftop area is generally done through a rooftop scaling factor which is a proxy for loss of rooftop area due to orientation, slope, and roof superstructures like chimneys, etc. Although, some studies exist at the country level where the rooftop scaling factor is documented, on a global scale no authoritative dataset exists that can demarcate country-wise rooftop scaling factors. Further work is required to document the country-specific rooftop scaling factors which are outside the scope of the research aim of this study.

Our cost assumptions cover 17 different countries across continents with average values for the rest of the countries. These assumptions can assign increased or decreased LCOE values to a certain region like Africa and South America. Another limitation of the cost assumptions is the inability of the LCOE metric to capture intra country variation of LCOE to a high degree due to lack of high-resolution cost data. Also, cost variability due to additional grid rollout, tariff mechanisms, and global change in prices due to trade protectionism practices are beyond the scope of the current assessment. Finally, we calculated all the cost and potential metrics assuming that no installed capacity exists for the ROI, where in the present time horizon, some installed capacity does exist.

A majority of limitations can be attributed to the underlying data used in our assessment, which can be improved with subsequent advancement in the methodology of the data providers. Further research can be undertaken to reduce the methodological limitations that are currently bootstrapped by the data availability and lack of homogenous global data.

## Supplementary information

Supplementary Information

## Data Availability

The data that support the findings of this study are available from the corresponding author upon reasonable request. The global road map is based on OpenStreetMap (OSM), which can be freely downloaded. The planet file used in this study is downloaded on April 1, 2020. The landcover map is based on Copernicus Global Land Service: Land Cover 100 m: collection 3: epoch 2015: Globe (10.5281/zenodo.2583745). Other data sources that are free to use are provided in the main text and in the “Methods” section.

## Code availability

Pseudocode to undertake this analysis can be found in the supplementary material (Supplementary Note 1) and should be read in conjunction with the “Methods” section. Base XGBoost Model is available at https://xgboost.readthedocs.io. The python script for plotting and data aggregation is available from the corresponding author upon reasonable request.
