# Supplementary material for: High resolution global spatiotemporal assessment of rooftop solar photovoltaics potential for renewable electricity generation
Source: Nat Commun. 2021 Oct 5;12:5738. doi: 10.1038/s41467-021-25720-2 (PMC8492708; doi:10.1038/s41467-021-25720-2)
Supplement: Supplementary file 1 — Supplementary Information [file 41467_2021_25720_MOESM1_ESM.pdf]

SUPPLEMENTARY INFORMATION

High resolution global spatiotemporal assessment of rooftop solar photovoltaic's potential for renewable electricity generation

Siddharth Joshi[1,2,3*], Shivika Mittal[4], Paul Holloway[2,5], Priyadarshi Ramprasad Shukla[6], Brian O'Gallachoir[1,2,3] , and James Glynn[1,2,3,7]

1 SFI MaREI Centre for Energy Climate and Marine, Ireland, 2 Environmental Research Institute, University College Cork, Ireland, 3 School of Engineering, University College Cork, Ireland, 4 Grantham Institute – Climate Change and the Environment, Imperial College London, United Kingdom, 5 Department of Geography, University College Cork, Ireland. 6 Global Centre for Environment and Energy, Ahmedabad University, India. 7 Center on Global Energy Policy, Columbia University, New York, USA

* e-mail: siddharth.joshi@ucc.ie

Supplementary Tables

Supplementary Table 1 | Distribution of sample FNs

World Regions	Countries	Building Footprint (km²)	Number of sample FNs
NORTH AMERICA	13	29,343	112,574
AFRICA	48	6,337	53,142
EUROPE	35	1,070	2,724
ASIA	39	333	696
AUSTRALIA	2	17	55
SOUTH AMERICA	10	15	79

Supplementary Table 2 | Input big datasets and links to validation studies

Datasets	Resolution	Usage	Validation
Building Footprints	Vector Dataset	Calculation of aggregated ground truth rooftop area	Heris, M.P., Foks, N.L., Bagstad, K.J. *et al.* A rasterized building footprint dataset for the United States. *Sci Data* **7,** 207 (2020). **https://doi.org/10.1038/s41597-020-0542-3**
Population	100 m²	Proxy for building density	Lloyd, C., Sorichetta, A. & Tatem, A. High resolution global gridded data for use in population studies. *Sci Data* **4,** 170001 (2017). **https://doi.org/10.1038/sdata.2017.1**
Road	Vector dataset	Proxy for built-up area density	Barrington-Leigh, C., & Millard-Ball, A. (2017). The world's user-generated road map is more than 80% complete. *PloS one*, *12*(8), e0180698. **https://doi.org/10.1371/journal.pone.0180698**
Conversion Factor	1 km²	Conversion of rooftop area to electriricy generation potential	ESMAP. 2019. Validation Report: Global Solar Atlas 2.0 Validation Report. Washington, DC: World Bank.
Land Cover	100 m²	Built-up area extent	Tsendbazar, N.E., Tarko, A., Linlin, L., Herold, M., Lesiv, M., Fritz, S., Maus. V; (2020): Copernicus Global Land Service: Land Cover 100m: Version 3 Globe 2015-2019: Validation Report; Zenodo, Geneve, Switzerland, September 2020; **doi: 10.5281/zenodo.3938974**

Supplementary Table 3 | Model Comparison

			10 fold Cross validation			Predicted Rooftop Area for Select Countries (km²)							
Model Name	Dependent Variable	Independent Variable	MSE	RMSE	R^2	220 countries	Ireland	France	Germany	Spain	UK	USA	India
Linear Regression	BFE	BA, RL, PPLN	0.03613	0.18912	0.957	198,826	182	4,567	4,820	1,959	2,530	27,440	12,605
Random Forest	BFE	BA, RL, PPLN	0.02593	0.16090	0.984	194,798	209	4,036	4,224	1,847	2,321	27,553	11,468
XGBoost	BFE	BA, RL, PPLN	0.02519	0.15837	0.981	193,910	218	3,938	4,164	1,851	2,400	27,585	11,731

Note: BFE is *estimated aggregated rooftop area per FN*, BA is *built-up area per FN*, RL is *aggregated road length per FN*, PPLN is *aggregated population count per FN.*

Supplementary Table 4 | Assessed global RTSPV potential in TWh/Year for combinations of scaling factor and panel efficiency [a]

Rooftop Scaling (%)	10% Efficiency	11% Efficiency	12% Efficiency	13% Efficiency	14% Efficiency	15% Efficiency	16% Efficiency	17% Efficiency	18% Efficiency	19% Efficiency	20% Efficiency
100	27,512	30,263	33,014	35,766	38,517	41,268	44,019	46,770	49,522	52,273	55,024
90	24,761	27,237	29,713	32,189	34,665	37,141	39,617	42,093	44,569	47,046	49,522
80	22,010	24,211	26,412	28,612	30,813	33,014	35,215	37,416	39,617	41,818	44,019
70	19,258	21,184	23,110	25,036	26,962	28,888	30,813	32,739	34,665	36,591	38,517
60	16,507	18,158	19,809	21,459	23,110	24,761	26,412	28,062	29,713	31,364	33,014
50	13,756	15,132	16,507	17,883	19,258	20,634	22,010	23,385	24,761	26,136	27,512
40	11,005	12,105	13,206	14,306	15,407	16,507	17,608	18,708	19,809	20,909	22,010
30	8,254	9,079	9,904	10,730	11,555	12,380	13,206	14,031	14,856	15,682	16,507
20	5,502	6,053	6,603	7,153	7,703	8,254	8,804	9,354	9,904	10,455	11,005
10	2,751	3,026	3,301	3,577	3,852	4,127	4,402	4,677	4,952	5,227	5,502

[a] Assuming 27,512 TWh per year global assessed RTSPV potential at 100% rooftop scaling factor scaling and 10% panel efficiency

Supplementary Table 5 | Change in Regional potentials with different combinations of panel efficiency and Rooftop Scaling Factor

32 World	Potential [a]	Eff_12_ RS_80	Eff_12_ RS_60	Eff_12_ RS_40	Eff_12_ RS_20	Eff_14_ RS_80	Eff_14_ RS_60	Eff_14_ RS_40	Eff_14_ RS_20	Eff_16_ RS_80	Eff_16_ RS_60	Eff_16_ RS_40	Eff_16_ RS_20	Eff_18_ RS_80	Eff_18_ RS_60	Eff_18_ RS_40	Eff_18_ RS_20
AFE	214	205	154	103	51	240	180	120	60	274	205	137	68	308	231	154	77
AFN	592	568	426	284	142	663	497	332	166	758	568	379	189	852	639	426	213
AFS	413	396	297	198	99	463	347	231	116	529	396	264	132	595	446	297	149
AFW	1,317	1,264	948	632	316	1,475	1,106	738	369	1,686	1,264	843	421	1,896	1,422	948	474
ANZ	404	388	291	194	97	452	339	226	113	517	388	259	129	582	436	291	145
ARG	285	274	205	137	68	319	239	160	80	365	274	182	91	410	308	205	103
ASC	748	718	539	359	180	838	628	419	209	957	718	479	239	1,077	808	539	269
ASE	1,354	1,300	975	650	325	1,516	1,137	758	379	1,733	1,300	867	433	1,950	1,462	975	487
ASO	304	292	219	146	73	340	255	170	85	389	292	195	97	438	328	219	109
ASR	219	210	158	105	53	245	184	123	61	280	210	140	70	315	237	158	79
BRA	997	957	718	479	239	1,117	837	558	279	1,276	957	638	319	1,436	1,077	718	359
CAN	327	314	235	157	78	366	275	183	92	419	314	209	105	471	353	235	118
CHN	4,375	4,200	3,150	2,100	1,050	4,900	3,675	2,450	1,225	5,600	4,200	2,800	1,400	6,300	4,725	3,150	1,575
ENE	499	479	359	240	120	559	419	279	140	639	479	319	160	719	539	359	180
ENW	75	72	54	36	18	84	63	42	21	96	72	48	24	108	81	54	27
EUE	648	622	467	311	156	726	544	363	181	829	622	415	207	933	700	467	233
EUW	2,210	2,122	1,591	1,061	530	2,475	1,856	1,238	619	2,829	2,122	1,414	707	3,182	2,387	1,591	796
GBR	238	228	171	114	57	267	200	133	67	305	228	152	76	343	257	171	86
IDN	878	843	632	421	211	983	738	492	246	1,124	843	562	281	1,264	948	632	316
IND	1,815	1,742	1,307	871	436	2,033	1,525	1,016	508	2,323	1,742	1,162	581	2,614	1,960	1,307	653
IRN	341	327	246	164	82	382	286	191	95	436	327	218	109	491	368	246	123
JPN	1,044	1,002	752	501	251	1,169	877	585	292	1,336	1,002	668	334	1,503	1,128	752	376
KOR	201	193	145	96	48	225	169	113	56	257	193	129	64	289	217	145	72
LAM	805	773	580	386	193	902	676	451	225	1,030	773	515	258	1,159	869	580	290
MEA	462	444	333	222	111	517	388	259	129	591	444	296	148	665	499	333	166
MEX	720	691	518	346	173	806	605	403	202	922	691	461	230	1,037	778	518	259
ROW	35	34	25	17	8	39	29	20	10	45	34	22	11	50	38	25	13
RUS	941	903	678	452	226	1,054	790	527	263	1,204	903	602	301	1,355	1,016	678	339
SAU	169	162	122	81	41	189	142	95	47	216	162	108	54	243	183	122	61
TUR	265	254	191	127	64	297	223	148	74	339	254	170	85	382	286	191	95
USA	4,247	4,077	3,058	2,039	1,019	4,757	3,567	2,378	1,189	5,436	4,077	2,718	1,359	6,116	4,587	3,058	1,529
ZAF	374	359	269	180	90	419	314	209	105	479	359	239	120	539	404	269	135
**Total**	**27,516**	**26,415**	**19,812**	**13,208**	**6,604**	**30,818**	**23,113**	**15,409**	**7,704**	**35,220**	**26,415**	**17,610**	**8,805**	**39,623**	**29,717**	**19,812**	**9,906**

[a] Assuming 27,512 TWh per year global assessed RTSPV potential at 100% rooftop scaling factor scaling and 10% panel efficiency.
Note: Eff_X_RS_Y, where"X" is the panel efficiency and "Y" is the Rooftop Scaling Factor. Colour coded cells are the aggregated regional potentials considering the average panel efficiency and rooftop scaling factor that are present in the wider literature.

Supplementary Table 6 | XGBoost Parameters

**Parameters**	**Values**
Gamma	3
Learning Rate	0.0445008698564836
Max Depth	3
Min Child Weight	3
Estimators	540
Regulation Alpha	4
Booster	Gbtree
Objective	Reg:squarederror
Column Sample By Tree	1
Threads	12
Hypertuning Iterations	500
Cross Validation	10 Fold

Base Model Download : https://xgboost.readthedocs.io/en/latest/

Supplementary Table 7 | Result Validation

Meta Data		Comparison Study										Our Study				
Reference	Study Area	Methodology		Rooftop Attributes			Technicccal Attributes	Output Resolution		Outputs		Reported Outputs [f]		Scaled Outputs [g]		Note
		Methodology	Rooftop area calculation Model Basis	Inclination	Orientation	Scaling Factor	Panel Efficiency	Temporal	Spatial	Rooftop Area (km^2)	Potential ($TWh\ yr^{-1}$)	Rooftop Area (km^2)	Potential ($TWh\ yr^{-1}$)	Rooftop Area (km^2)	Potential ($TWh\ yr^{-1}$)	Camparison Metric
**GLOBAL/REGIONAL Level**																
1	EU [a]	Top Down	Statistical Scaling	NO	NO	0.30	N.A.	Yearly	100 m^2	7,935	680	25,323	NA	7,597	N.A.	Rooftop Area
2	Global [b]	Bottom Up	Gomprets Curve	NO	NO	0.32	14%	Yearly	0.5° × 0.5°/ 55 km^2	N.A.	8,300	193,875	27,512	N.A.	12,300	Aggregated Potential
3	Global [c]	Bottom Up	Statistical Scaling	NO	NO	0.33	13%	Yearly	Country	N.A.	8,611	193,875	27,512	N.A.	10,894	Aggregated Potential
4	179 countries [d]	Bottom Up	Statistical Scaling	YES	YES	0.42	20.40%	Yearly	Country	93,513	N.A.	193,875	N.A.	81,428	N.A.	Rooftop Area
5	17 Regions [e]	Bottom Up	Statistical Scaling	NO	NO	0.11	14%	Yearly	17 Regions	150,000	N.A.	193,875	N.A.	193,875	N.A.	Rooftop Area
**COUNTRY Level**																
Switzerland																
6	Switzerland	Top Down	Random Forest	YES	YES	0.94	13%	Monthly	200 m^2	252	17	370	N.A.	348	N.A.	Rooftop Area
7	Switzerland	Top Down	Random Forest	YES	YES	0.56	15%	Yearly	ROI aggregated	267±71	24±9	370	45	209	38	Rooftop Area and Aggregated Potential
4	179 countries	Bottom Up	Statistical Scaling	YES	YES	0.28	20.40%	Yearly	Country	127	N.A.	370	N.A.	104	N.A.	Rooftop Area
Spain																
8	Spain	Top Down	Stratified Sampling	NO	NO	0.20	N.A.	N.A.	N.A.	571±183	N.A.	1,851	N.A.	365	N.A.	Rooftop Area
1	Spain	Top Down	Statistical Scaling	NO	NO	0.30	N.A.	Yearly	100 m^2	492	65	1,851	294	555	N.A.	Rooftop Area
4	179 countries	Bottom Up	Statistical Scaling	YES	YES	0.36	20.40%	Yearly	Country	721	N.A.	1,851	N.A.	666	N.A.	Rooftop Area
USA																
9	USA	Hybrid	Statistical Scaling	YES	YES	0.32	14%	Yearly	Sub National	8,130	1,432	27,585	4,250	8,827	1,904	Rooftop Area and Aggregated Potential
2	USA	Bottom Up	Gomprets Curve	NO	NO	0.32	14%	Yearly	0.5° × 0.5°/ 55 km^2	2,637	600	N.A.	4,250	N.A.	2,050	Rooftop Area
4	179 countries	Bottom Up	Statistical Scaling	YES	YES	0.36	20.40%	Yearly	Country	10,178	N.A.	27,585	N.A.	9,931	N.A.	Rooftop Area
Germany																
10	Germany	Bottom Up	Building Type	NO	NO	0.27-0.58	12.00%	Yearly	Municipal	N.A.	148	4,163	450	N.A.	145	Aggregated Potential
1	Germany	Top Down	Statistical Scaling	NO	NO	0.30	N.A.	Yearly	100 m^2	1,523	104	4,163	450	1,249	189	Rooftop Area and Aggregated Potential
4	179 countries	Bottom Up	Statistical Scaling	YES	YES	0.26	20.40%	Yearly	Country	827	N.A.	4,163	N.A.	1,082	N.A.	Rooftop Area
France																
11	France	Bottom Up	Statistical Scaling	NO	NO	0.40	12.70%	N.A.	Countrywise	400	N.A.	3,938	N.A.	1,575	N.A.	Rooftop Area
1	France	Top Down	Statistical Scaling	NO	NO	0.30	N.A.	Yearly	100m^2	1,346	125	3,938	N.A.	1,181	N.A.	Rooftop Area
4	179 countries	Bottom Up	Statistical Scaling	YES	YES	0.29	20.40%	Yearly	Country	943	N.A.	3,938	N.A.	1,142	N.A.	Rooftop Area
UK																
11	UK	Bottom Up	Statistical Scaling	NO	NO	0.40	12.70%	N.A.	Countrywise	500	N.A.	2,400	N.A.	960	N.A.	Rooftop Area
1	UK	Top Down	Statistical Scaling	NO	NO	0.30	N.A.	Yearly	100 m^2	770	44	2,400	N.A.	720	N.A.	Rooftop Area
4	UK	Bottom Up	Statistical Scaling	YES	YES	0.20	20.40%	Yearly	Country	488	N.A.	2,400	N.A.	478	N.A.	Rooftop Area
Vietnam																
12	Vietnam	Bottom Up	Statistical Scaling	YES	NO	0.5-0.58	16.50%	Yearly	Provincial	1,402	278	2,400	306	1,320	278	Rooftop Area and Aggregated Potential
4	179 countries	Bottom Up	Statistical Scaling	YES	YES	0.55	20.40%	Yearly	Country	1,072	N.A.	2,400	N.A.	1,320	N.A.	Rooftop Area
Tanzania																
4	179 countries	Bottom Up	Statistical Scaling	YES	YES	0.71	20.40%	Yearly	Country	810	N.A.	795	N.A.	564	N.A.	Rooftop Area
Mozambique																
4	179 countries	Bottom Up	Statistical Scaling	YES	YES	0.56	20.40%	Yearly	Country	376	N.A.	428	N.A.	240	N.A.	Rooftop Area
Canada																
4	179 countries	Bottom Up	Statistical Scaling	YES	YES	0.25	20.40%	Yearly	Country	751	N.A.	2,394	N.A.	599	N.A.	Rooftop Area
China																
2	China	Bottom Up	Gomprets Curve	NO	NO	0.32	14%	Yearly	0.5° × 0.5°/ 55 km^2	N.A.	2,300	N.A.	4,375	N.A.	2,100	Aggregated Potential
4	179 countries	Bottom Up	Statistical Scaling	YES	YES	0.35	20.40%	Yearly	Country	11,983	N.A.	35,156	N.A.	12,305	N.A.	Rooftop Area
Russia																
2	Russia	Bottom Up	Gomprets Curve	NO	NO	0.32	14%	Yearly	0.5° × 0.5°/ 55 km^2	N.A.	450	N.A.	940	N.A.	451	Aggregated Potential
4	179 countries	Bottom Up	Statistical Scaling	YES	YES	0.23	20.40%	Yearly	Country	1,592	N.A.	8,038	N.A.	1,849	N.A.	Rooftop Area
Brazil																
4	179 countries	Bottom Up	Statistical Scaling	YES	YES	0.53	20.40%	Yearly	Country	4,411	N.A.	6,386	N.A.	3,385	N.A.	Rooftop Area
Angola																
4	179 countries	Bottom Up	Statistical Scaling	YES	YES	0.65	20.40%	Yearly	Country	416	N.A.	315	N.A.	205	N.A.	Rooftop Area
**City/ District Level**																
13	Gangnam District, Seoul, South Korea	Top Down	Hillshade Analysis	NO	YES	0.66	15%	Monthly	ROI aggregated	5	1	N.A.	1	N.A.	1	Aggregated Potential
14	Osaka	Top Down	Statistical Scaling	YES	YES	0.55-0.65	20%	Yearly	ROI	42±9	7	N.A.	8	N.A.	9	Aggregated Potential

[a] Study conducted for EU-27 countries including UK. We have used the same regional definition for our comparison

[b] Only residential sector covered in the study. We have compared all sectors here

[c] All sectors considered in the study

[d] We are comparing our results for 220 countries with 179 countries in the study

[e] Only global study to use GDP as a driver for rooftop area

[f] Origional values documented in our studies

[g] Scaled values after incorparating assumprions of the comparison study

Supplementary Table 8 | 8 Global World Region Mapping

World Regions	Country Name
AFRICA	Angola,Burundi,Benin,Burkina Faso,Botswana,Central African Republic,Côte d'Ivoire,Cameroon,Democratic Republic of the Congo,Republic of Congo,Comoros,Cape Verde,Djibouti,Algeria,Egypt,Eritrea,Western Sahara,Ethiopia,Gabon,Ghana,Guinea,Gambia,Guinea-Bissau,Equatorial Guinea,Kenya,Liberia,Libya,Lesotho,Morocco,Madagascar,Mali,Mozambique,Mauritania,Mauritius,Malawi,Mayotte,Namibia,Niger,Nigeria,Reunion,Rwanda,Sudan,Senegal,Saint Helena,Sierra Leone,Somalia,South Sudan,São Tomé and Príncipe,Swaziland,Seychelles,Chad,Togo,Tunisia,Tanzania,Uganda,South Africa,Zambia,Zimbabwe
ASIA	Afghanistan,United Arab Emirates,Armenia,Azerbaijan,Bangladesh,Bahrain,Brunei,Bhutan,China,Cyprus,Georgia,Hong Kong,Indonesia,India,Iran,Iraq,Israel,Jordan,Japan,Kazakhstan,Kyrgyzstan,Cambodia,South Korea,Kuwait,Laos,Lebanon,Sri Lanka,Macao,Maldives,Myanmar,Mongolia,Malaysia,New Caledonia,Nepal,Oman,Pakistan,Philippines,Papua New Guinea,North Korea,Palestina,Qatar,Russia,Saudi Arabia,Singapore,Solomon Islands,Syria,Thailand,Tajikistan,Turkmenistan,Timor-Leste,Turkey,Taiwan,Uzbekistan,Vietnam,Vanuatu,Yemen
AUSTRALIA	Australia,New Zealand
EUROPE	Kosovo,Albania,Andorra,Austria,Belgium,Bulgaria,Bosnia and Herzegovina,Belarus,Switzerland,Czech Republic,Germany,Denmark,Spain,Estonia,Finland,France,Faroe Islands,United Kingdom,Gibraltar,Greece,Croatia,Hungary,Isle of Man,Ireland,Iceland,Italy,Liechtenstein,Lithuania,Luxembourg,Latvia,Monaco,Moldova,Macedonia,Malta,Montenegro,Netherlands,Norway,Poland,Portugal,Romania,San Marino,Serbia,Slovakia,Slovenia,Sweden,Ukraine,Vatican City
ISLAND NATIONS	American Samoa,Cook Islands,Fiji,Micronesia,Guam,Kiribati,Marshall Islands,Northern Mariana Islands,Niue,Nauru,Palau,French Polynesia,Tokelau,Tonga,Tuvalu,Wallis and Futuna,Samoa
NORTH AMERICA	Aruba,Antigua and Barbuda,Bonaire, Sint Eustatius and Saba,Bahamas,Belize,Bermuda,Barbados,Canada,Costa Rica,Cuba,Curaçao,Cayman Islands,Dominica,Dominican Republic,Guadeloupe,Grenada,Greenland,Guatemala,Honduras,Haiti,Jamaica,Saint Kitts and Nevis,Saint Lucia,Mexico,Montserrat,Martinique,Nicaragua,Panama,Puerto Rico,El Salvador,Saint Pierre and Miquelon,Turks and Caicos Islands,Trinidad and Tobago,United States,Saint Vincent and the Grenadines,British Virgin Islands,Virgin Islands, U.S.
ROW	Anguilla,Saint-Barthélemy,Saint-Martin,Sint Maarten
SOUTH AMERICA	Argentina,Bolivia,Brazil,Chile,Colombia,Ecuador,Falkland Islands,French Guiana,Guyana,Peru,Paraguay,Suriname,Uruguay,Venezuela

Supplementary Table 9 | CAPEX mapping

CAPEX Mappings	Country Name
AUS	Australia,New Caledonia,New Zealand,Palau,Solomon Islands,Tonga,Vanuatu
BRA	Argentina,Bahamas,Belize,Bolivia,Brazil,Chile,Colombia,Costa Rica,Cuba,Dominican Republic,Ecuador,Falkland Islands,Guatemala,French Guiana,Guyana,Honduras,Haiti,Jamaica,Mexico,Nicaragua,Panama,Peru,Puerto Rico,Paraguay,El Salvador,Suriname,Uruguay,Venezuela
CHE	Switzerland
CHN	China
DEU	Germany
ESP	Spain
FRA	France
GBR	United Kingdom
IND	India
ITA	Italy,San Marino,Vatican City
JPN	Japan
KOR	South Korea
MYS	Malaysia
THA	Thailand
USA	Canada,United States
ZAF	Lesotho,South Africa
EU_AVG	Kosovo,Albania,Andorra,Armenia,Austria,Azerbaijan,Belgium,Bulgaria,Bosnia and Herzegovina,Belarus,Cyprus,Czech Republic,Denmark,Estonia,Finland,Georgia,Greece,Croatia,Hungary,Isle of Man,Ireland,Iceland,Liechtenstein,Lithuania,Luxembourg,Latvia,Monaco,Moldova,Macedonia,Malta,Montenegro,Netherlands,Norway,Poland,Portugal,Romania,Serbia,Slovakia,Slovenia,Sweden,Ukraine
ASIA_AVG	Afghanistan,United Arab Emirates,Bangladesh,Bahrain,Brunei,Bhutan,Hong Kong,Indonesia,Iran,Iraq,Israel,Jordan,Kazakhstan,Kyrgyzstan,Cambodia,Kuwait,Laos,Lebanon,Sri Lanka,Macao,Myanmar,Mongolia,Nepal,Oman,Pakistan,Philippines,Papua New Guinea,North Korea,Palestina,Qatar,Russia,Saudi Arabia,Singapore,Syria,Tajikistan,Turkmenistan,Timor-Leste,Turkey,Taiwan,Uzbekistan,Vietnam,Yemen
WLD_AVG	Aruba,Angola,Anguilla,American Samoa,Antigua and Barbuda,Burundi,Benin,Bonaire, Sint Eustatius and Saba,Burkina Faso,Saint-Barthélemy,Bermuda,Barbados,Botswana,Central African Republic,Côte d'Ivoire,Cameroon,Democratic Republic of the Congo,Republic of Congo,Cook Islands,Comoros,Cape Verde,Curaçao,Cayman Islands,Djibouti,Dominica,Algeria,Egypt,Eritrea,Western Sahara,Ethiopia,Fiji,Faroe Islands,Micronesia,Gabon,Ghana,Guinea,Guadeloupe,Gambia,Guinea-Bissau,Equatorial Guinea,Grenada,Greenland,Guam,Kenya,Kiribati,Saint Kitts and Nevis,Liberia,Libya,Saint Lucia,Saint-Martin,Morocco,Madagascar,Maldives,Marshall Islands,Mali,Northern Mariana Islands,Mozambique,Mauritania,Montserrat,Martinique,Mauritius,Malawi,Mayotte,Namibia,Niger,Nigeria,Niue,Nauru,French Polynesia,Reunion,Rwanda,Sudan,Senegal,Saint Helena,Sierra Leone,Somalia,Saint Pierre and Miquelon,South Sudan,São Tomé and Príncipe,Swaziland,Sint Maarten,Seychelles,Turks and Caicos Islands,Chad,Togo,Tokelau,Trinidad and Tobago,Tunisia,Tuvalu,Tanzania,Uganda,Saint Vincent and the Grenadines,British Virgin Islands,Virgin Islands, U.S.,Wallis and Futuna,Samoa,Zambia,Zimbabwe

Supplementary Table 10 | 32 Global Region Mapping

32 Regions	Country Name
AFE	Eritrea,Ethiopia,Kenya,Mauritius,Sudan,South Sudan
AFN	Algeria,Egypt,Libya,Morocco,Tunisia
AFS	Angola,Botswana,Mozambique,Namibia,Tanzania,Zambia,Zimbabwe
AFW	Burundi,Benin,Burkina Faso,Central African Republic,Côte d'Ivoire,Cameroon,Democratic Republic of the Congo,Republic ofCongo, Comoros,Cape Verde,Djibouti,Gabon,Ghana,Guinea,Gambia,Guinea-Bissau,Equatorial Guinea,Liberia,Lesotho,Madagascar,Mali, Mauritania,Malawi,Niger,Nigeria,Reunion,Rwanda,Senegal,Sierra Leone,Somalia,São Tomé and Príncipe,Swaziland,Seychelles, Chad,Togo,Uganda
ANZ	Australia,New Zealand
ARG	Argentina
ASC	Armenia,Azerbaijan,Georgia,Kazakhstan,Kyrgyzstan,Mongolia,Pakistan,Tajikistan,Turkmenistan,Uzbekistan
ASE	Brunei,Cambodia,Myanmar,Malaysia,Philippines,Singapore,Thailand,Vietnam
ASO	Bangladesh,Sri Lanka,Nepal
ASR	Afghanistan,Bhutan,Cook Islands,Fiji,Hong Kong,Kiribati,Laos,Macao,Maldives,New Caledonia,Palau,Papua New Guinea,North Korea,French Polynesia,Solomon Islands,Timor-Leste,Tonga,Taiwan,Vanuatu,Samoa
BRA	Brazil
CAN	Canada
CHN	China
ENE	Albania,Bosnia and Herzegovina,Belarus,Moldova,Macedonia,Montenegro,Serbia,Ukraine
ENW	Switzerland,Iceland,Norway
EUE	Bulgaria,Cyprus,Czech Republic,Estonia,Croatia,Hungary,Lithuania,Latvia,Malta,Poland,Romania,Slovakia,Slovenia
EUW	Austria,Belgium,Germany,Denmark,Spain,Finland,France,Greece,Ireland,Italy,Luxembourg,Netherlands,Portugal,Sweden
GBR	United Kingdom,Gibraltar
IDN	Indonesia
IND	India
IRN	Iran
JPN	Japan
KOR	South Korea
LAM	Aruba,Antigua and Barbuda,Bonaire, Sint Eustatius and Saba,Bahamas,Belize,Bermuda,Bolivia,Barbados,Chile,Colombia,Costa Rica,Cuba,Curaçao,Cayman Islands,Dominica,Dominican Republic,Ecuador,Falkland Islands,Guadeloupe,Grenada,Guatemala,French Guiana,Guyana,Honduras,Haiti,Jamaica,Saint Kitts and Nevis,Saint Lucia,Montserrat,Martinique,Nicaragua,Panama,Peru,Puerto Rico,Paraguay,El Salvador,Saint Pierre and Miquelon,Suriname,Sint Maarten,Turks and Caicos Islands,Trinidad and Tobago,Uruguay,Saint Vincent and the Grenadines,Venezuela,British Virgin Islands
MEA	United Arab Emirates,Bahrain,Iraq,Israel,Jordan,Kuwait,Lebanon,Oman,Qatar,Syria,Yemen
MEX	Mexico
ROW	Kosovo,Anguilla,Andorra,American Samoa,Saint-Barthélemy,Western Sahara,Faroe Islands,Micronesia,Greenland,Guam,Isle of Man,Liechtenstein,Saint-Martin,Monaco,Marshall Islands,Northern Mariana Islands,Mayotte,Niue,Nauru,Palestina,Saint Helena,San Marino,Tokelau,Tuvalu,Vatican City,Virgin Islands, U.S.,Wallis and Futuna
RUS	Russia
SAU	Saudi Arabia
TUR	Turkey
USA	United States
ZAF	South Africa

Supplementary Figures

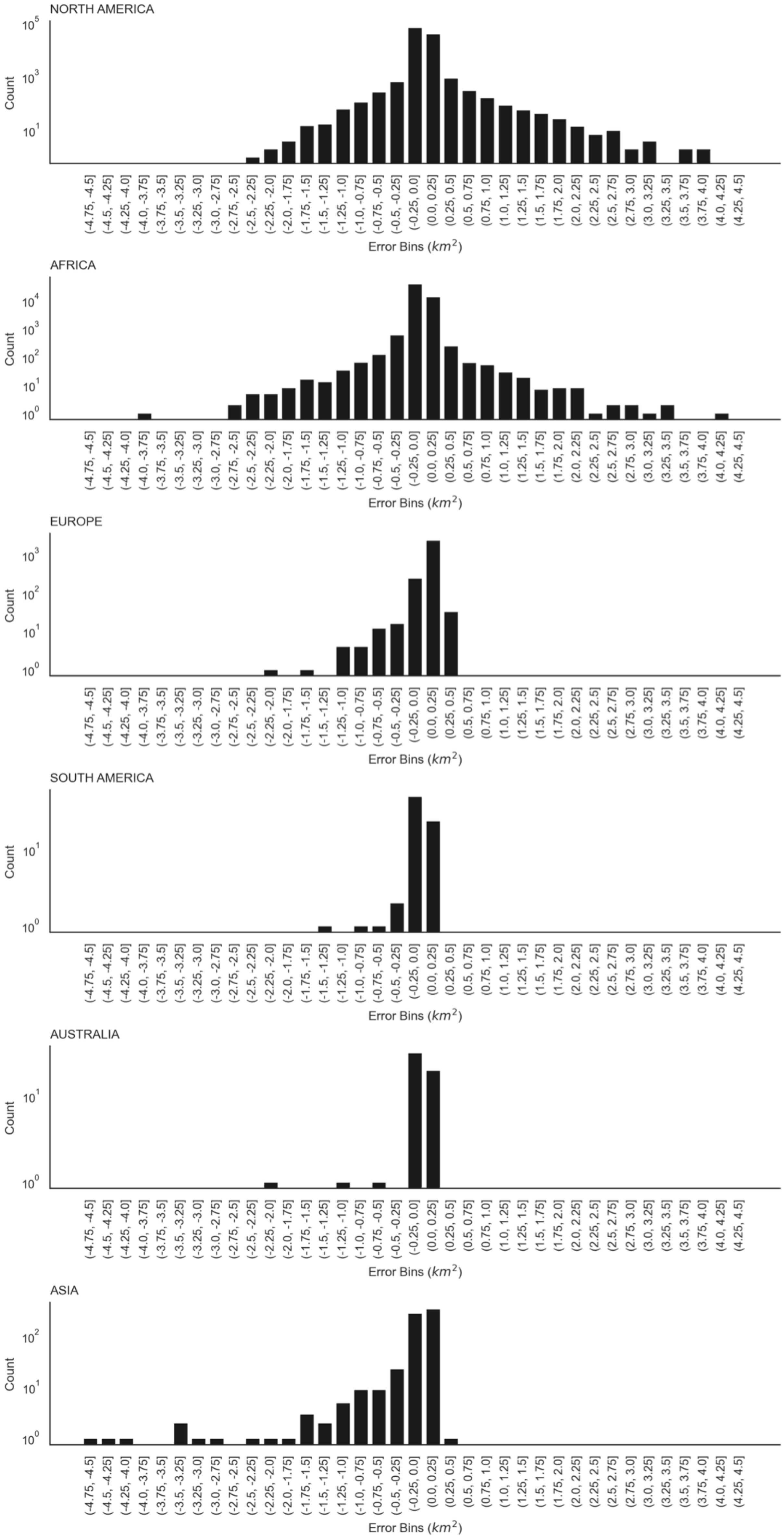

Supplementary Figure 1 | Distribution of error bins at a regional level

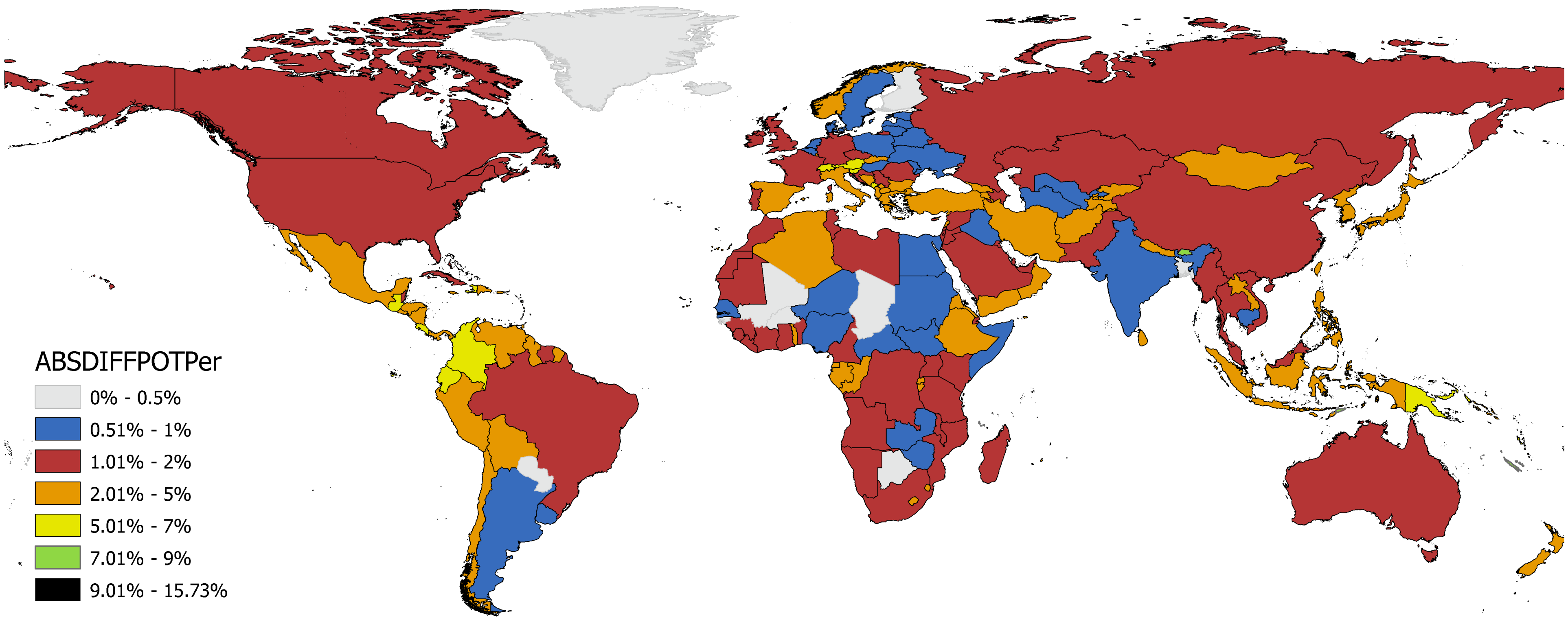

Supplementary Figure 2 | Absolute percentage difference in potential

The figure is a author generated visualisation of data representing absolute percentage difference in yearly potentials when using either maximum or minimum CF factor value inside an FN compared to mean statistic used in the study. Majority of the global countries have an absolute percentage difference between 0-2% when compared to their mean yearly potential. The countries having high absolute percentage difference are the ones that are located in a mountainous terrain. The range of the absolute percentage difference are the bounds of uncertainty for a country, within which the actual value is expected to fall depending on whether the FN having the deviation contains built-up area or not. On a global aggregated level, an absolute difference of ±1% is observed around mean yearly potential of 27 PWh.

Country Mappings (Figures 3a-d):

Following are the geographical locations of various regions and mappings used in the research.

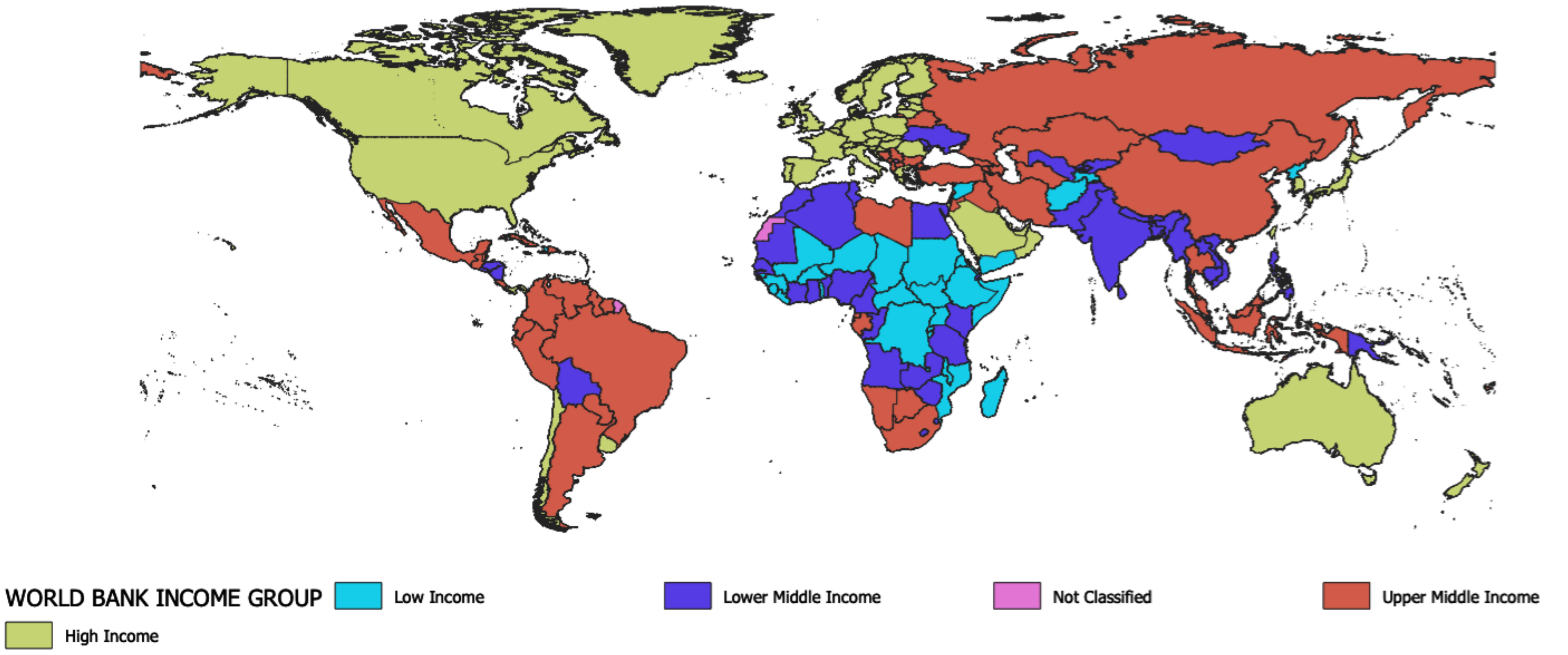

Supplementary Figure 3a | World Bank Income Groups For the current 2021 fiscal year, low-income economies are defined as those with a GNI per capita, calculated using the World Bank Atlas method, of $1,035 or less in 2019; lower middle-income economies are those with a GNI per capita between $1,036 and $4,045; upper middle-income economies are those with a GNI per capita between $4,046 and $12,535; high-income economies are those with a GNI per capita of $12,536 or more. Mapping Data Source: World Bank, visualisation done by author.

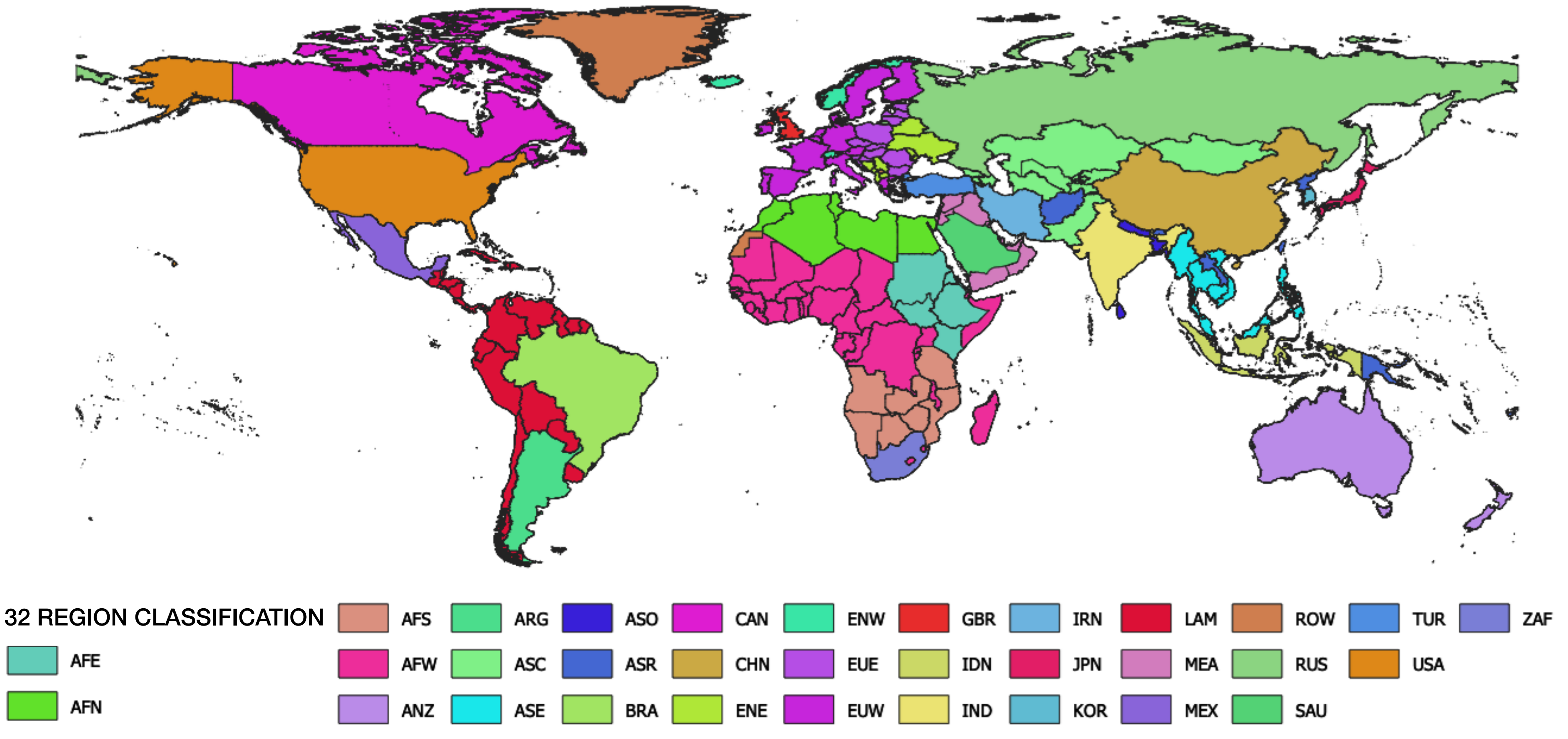

Supplementary Figure 3b | 32 Region global classification. The 32 region global map is a visualisation by author based on the regions description of CHIMERA project that is being executed as a collaboration project between University College Cork, Ireland, Tshinghua University, China and Peking University, China to design next generation global energy models with high temporal and spatial resolution. The regions are designed based on geographic continuity and sub regional uniqueness. These regions will act as a base unit for further work being done under the project.

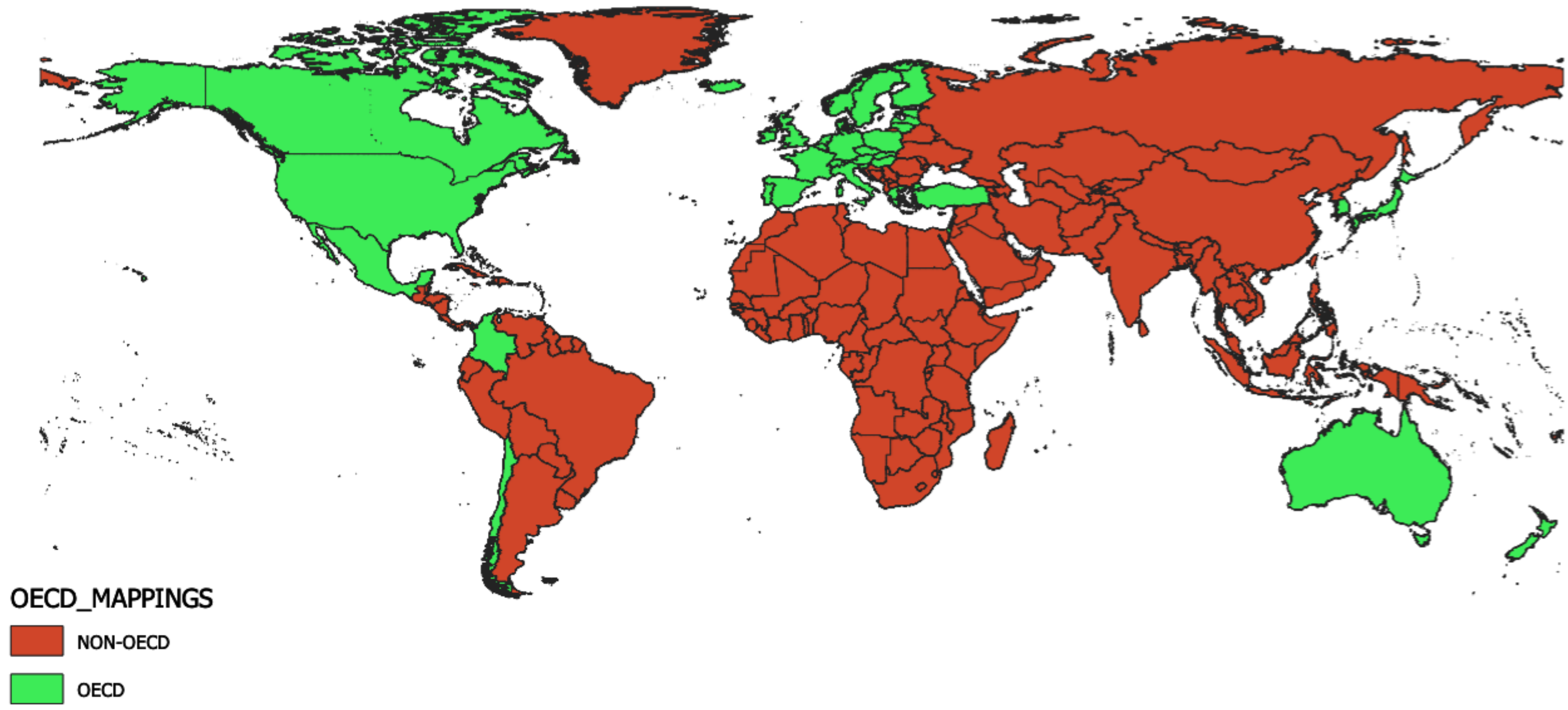

Supplementary Figure 3c | OECD country classification The OECD classification is based on the global charter of Organisation for Economic Co-operation and Development group. The OECD classification is used to map OPEX and Discount Rates for the current research. Mapping Data Source: OECD, visualisation done by author.

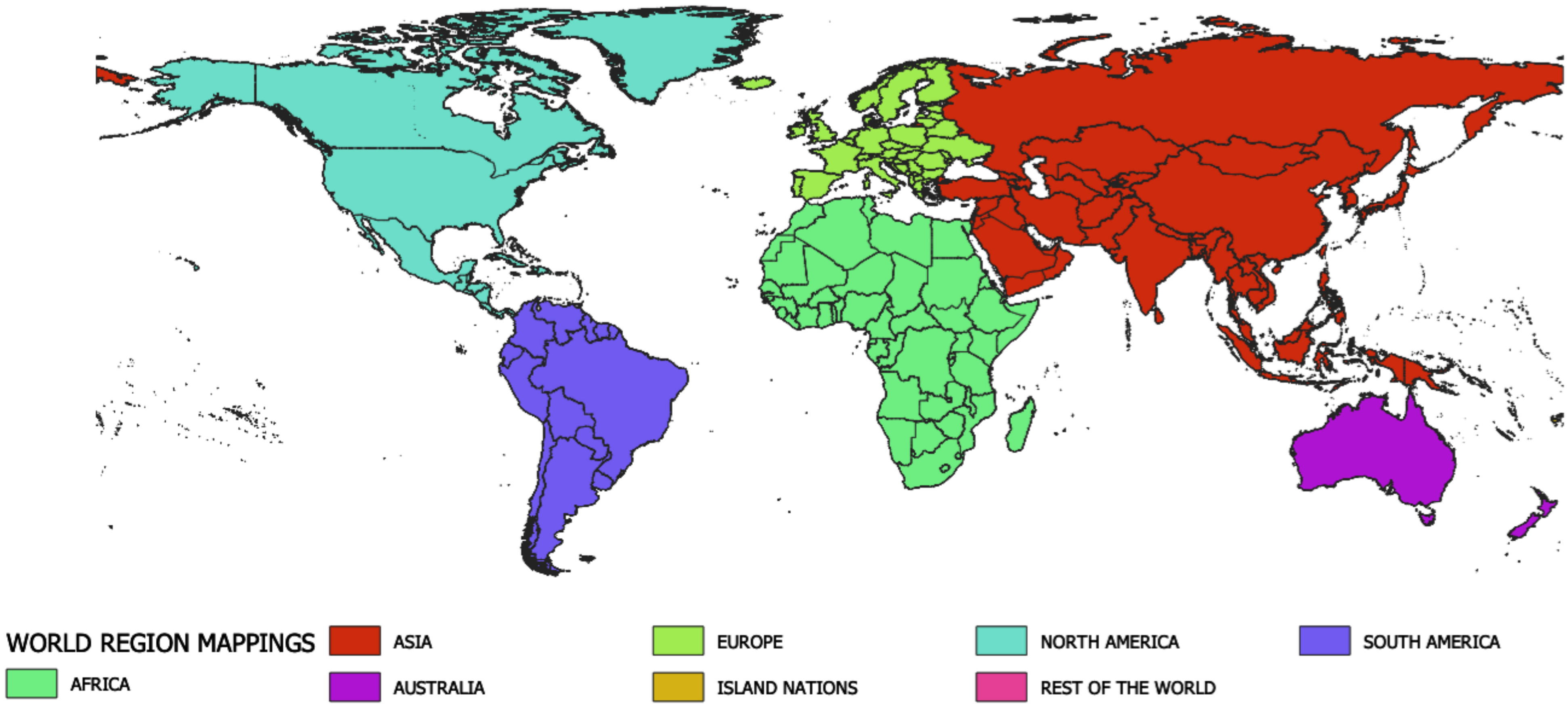

Supplementary Figure 3d | World Regions The world region classification is an author generated visualisation based off assigning the countries to respective global continent with exception of Cyprus and Russia, which is assigned to Asia continent. The Island Nations region is assigned to small group of Island countries in Asia-Pacific region.

Supplementary Note 1: Pseudocode for analysis framework

Algorithm 1: Data Preparation

Input:

- **FN:** Fishnet Grid
- **BF:** Building Footprint polygon (Vector)
- **RL:** Road polyline (Vector)
- **BA:** Built-up Area (Raster)
- **CF_M:** Monthly Conversion Factor (Raster)
- **PPLN:** Population count (Raster)

Output:

- **BF_{FN}:** Aggregated Building Footprint area mapped to unique FN
- **RL_{FN}:** Aggregated Road Length mapped to unique FN
- **BA_{FN}:** Aggregated Built-up Area mapped to unique FN
- **$CF_{M,FN}$:** Averaged Monthly Conversion Factor mapped to unique FN
- **$PPLN_{FN}$:** Aggregated Population count mapped to unique FN

for each FN: /* Iterate for each cell in Fishnet Grid*/
  if BA>0: /* If built-up area exist in FN*/
    $MASK_{FN}$ = **not** (Geographic extent of BA in FN) /* Generate Masking layer*/
    BF_{FN} = **sum** (Area of BF outside $MASK_{FN}$) /* Aggregate non-masked Building Footprint area*/
    RL_{FN} = **sum** (Length of RL outside $MASK_{FN}$) /* Aggregate non-masked length of Roads*/
    $PPLN_{FN}$ = **sum** (Pixels of PPLN outside $MASK_{FN}$) /* Aggregate non-masked Population count*/
    BA_{FN} = **sum** (PixelValue*PixelArea of BA)/100 /* Aggregate non-masked Built-up area*/
    $CF_{M,FN=}$= **mean** (Pixels of CF_M) /* Average of Conversion Factor value for each month*/

Algorithm 2: XGBoost Model Training and Estimation

Input:

BF_{FN}: Aggregated Building Footprint area mapped to unique FN
RL_{FN}: Aggregated Road Length mapped to unique FN
BA_{FN}: Aggregated Built-up Area mapped to unique FN
$PPLN_{FN}$: Aggregated Population count mapped to unique FN
S: Denotes samples extracted from full data

Output:

BFE_{FN}: Aggregated estimated Building Footprint area mapped to unique FN

# Sample Fishnet Cell extraction

for each FN: /* Iterate for each cell in Fishnet Grid*/
  if $BF_{FN} > 0$: /* If Aggregated Building Footprint area exist in FN*/
    FNs = FN /* FNs becomes sample FN for model training*/

# Hyper-parameter Optimisation (Aim is to reduce Mean Squared Error of the base model with each iteration)

for n: /* n is the number of iterations*/
  $Xs = BF_{FNs}$ /* Set dependent variable for sample FNs */
  $Ys = (RL_{FNs}, BA_{FNs}, PPLN_{FNs})$ /* Set independent variables dataframe for sample FNs */
  $Ks_i = Xs_i, Ys_i$ /* Split data into 10 folds using shuffling, *i* is the ith fold */
  MSE_i = **XGB_P** (Ks_i) /* Calculate MSE for i^{th} fold data using "P" parameters for base XGBoost model*/
  $MSE_{mean,t}$ = **mean** ($MSE_{1\text{-}10}$) /*Take mean of MSE for all 10 folds at t iteration number*/
  if $MSE_{mean,t-1} > MSE_{mean,t}$: /*If MSE of *t-1* iteration number is greater than MSE of *t* iteration*/
    Choose the next best parameter combination based on predictive *Tree Parzen Estimator*
save P /*Save best parameter combination after *n* iterations*/

# Estimation of Building Footprint Area per FN from trained model

$Xs = BF_{FNs}$ /* Set dependent variable for sample FNs */
$Ys = (RL_{FNs}, BA_{FNs}, PPLN_{FNs})$ /* Set independent variables dataframe for sample FNs */
Model = **XGB_P** (X_s, Y_s) /* Train base XGBoost model using tuned parameters*/
$Y = (RL_{FN}, BA_{FN}, PPLN_{FN})$ /* Set independent variables dataframe for full set of FN */
if RL_{FN} **and** BA_{FN} **and** $PPLN_{FN}$ **is not null**: /* If logical data exists within the FN */
  BFE_{FN}= **Model** (Y) /*Use trained model to estimate aggregated building footprint area per FN */
save BFE_{FN} /*Save estimated Building footprint data for FN*/

Algorithm 3: Calculation of Potentials and Costs

Input:

- **BFE_{FN}:** Aggregated estimated Building Footprint area mapped to unique FN
- **$CF_{M,FN}$:** Averaged Monthly Conversion Factor mapped to unique FN
- **A_P:** Area required by each solar panel
- **RAF:** Rooftop Availability Factor
- **$CAPEX_{FN}$:** Aggregated Capital Expenditure mapped to unique FN
- **$OPEX_{FN}$:** Aggregated Operation and Maintenance cost per year mapped to unique FN
- **DR_{FN}:** Average Discount Rate mapped to unique FN
- **TL** : Technology life of Rooftop Solar PV

Output:

- **$SP_{M,FN}$:** Estimated monthly electricity generation potential mapped to unique FN
- **$SP_{Y,FN}$:** Estimated yearly electricity generation potential mapped to unique FN
- **$LCOE_{FN}$:** Estimated Levelised Cost of Electricity mapped to unique FN

# Calculation of potential per FN cell

for each FN: /* Iterate for each cell in Fishnet Grid*/
  for each M: /* Iterate for each month*/
    if BFE_{FN}>0: /* If estimated aggregated Building Footprint area exist in FN*/
      $NP_{FN} = (BFE_{FN}*RAF)/A_P$ /*calculate number of 1 kW_p panels per FN*/
      $SP_{M,FN} = NP_{FN}*CF_{M,FN}$ /*Calculate monthly potential per FN*/
$SP_{Y,FN}$ = **sum**($SP_{M,FN}$) /*Calculate yearly potential per FN*/
save $SP_{Y,FN}$,$SP_{M,FN}$ /*save $SP_{Y,FN}$,$SP_{M,FN}$ as Geopackage and csv file*/
create Figure 5a using $SP_{Y,FN}$ /*Use geomapped $SP_{Y,FN}$ to create figure 5a */

# Calculation of LCOE per FN cell

for each FN: /* n is the number of iterations*/
    map CAPEX, OPEX and DR /* Map CAPEX, OPEX and DR based on FN geolocation */
    $CAPEX_{FN}$ = CAPEX* NP_{FN} /* Find total CAPEX using number of panels */
    $OPEX_{FN}$ = OPEX* NP_{FN} /* Find total OPEX using number of panels */
    $LCOE_{FN}$=**function**($CAPEX_{FN}$, $OPEX_{FN}$, DR_{FN}, $SP_{Y,FN}$,TL) /* Calculate LCOE */
save $LCOE_{FN}$ /*save $LCOE_{FN}$ as Geopackage and csv file*/
create Figure 5b using $LCOE_{FN}$ /*Use geomapped $LCOE_{FN}$ to create figure 5b */

Supplementary references

1 Bódis, K., et al. (2019). "A high-resolution geospatial assessment of the rooftop solar photovoltaic potential in the European Union." Renewable and Sustainable Energy Reviews 114.

2 Gernaat, D. E. H. J., et al. (2020). "The role of residential rooftop photovoltaic in long-term energy and climate scenarios." Applied Energy 279.

3 Deng, Y. Y. et al. (2015) Quantifying a realistic, worldwide wind and solar electricity supply. Global Environmental Change 31, 239–252 (2015).

4 Jacobson, M. Z., et al. (2017). "100% Clean and Renewable Wind, Water, and Sunlight All-Sector Energy Roadmaps for 139 Countries of the World." Joule 1(1): 108-121.

5 Hoogwijk, M., 2004. On the global and regional potential of renewable energy sources. Ph.D. Thesis, Universiteit Utrecht, Utrecht.

6 Assouline, D., et al. (2018). "Large-scale rooftop solar photovoltaic technical potential estimation using Random Forests." Applied Energy 217: 189-211.

7 Walch, A., et al. (2020). "Big data mining for the estimation of hourly rooftop photovoltaic potential and its uncertainty." Applied Energy 262.

8 Izquierdo, S., et al. (2008). "A method for estimating the geographical distribution of the available roof surface area for large-scale photovoltaic energy-potential evaluations." Solar Energy 82(10): 929-939.

9 Gagnon, P., et al. (2018). "Estimating rooftop solar technical potential across the US using a combination of GIS-based methods, lidar data, and statistical modeling." Environmental Research Letters 13(2).

10 Mainzer, K., et al. (2014). "A high-resolution determination of the technical potential for residential-roof-mounted photovoltaic systems in Germany." Solar Energy 105: 715-731.

11 Defaix, P. R., et al. (2012). Technical potential for photovoltaics on buildings in the EU-27. Solar Energy 86, 2644–2653.

12 Minh Khuong, P., et al. (2020). "A Cost-Effective and Transferable Methodology for Rooftop PV Potential Assessment in Developing Countries." Energies 13(10).

13 Hong, T., et al. (2017). "Development of a method for estimating the rooftop solar photovoltaic (PV) potential by analyzing the available rooftop area using Hillshade analysis." Applied Energy 194: 320-332.

14 Yuan, J., et al. (2016). "A method to estimate the potential of rooftop photovoltaic power generation for a region." Urban Climate 17: 1-19.
